# 4D Assembly of Time‐dependent Lanthanide Supramolecular Multicolor Phosphorescence for Encryption and Visual Sensing

**DOI:** 10.1002/advs.202415418

**Published:** 2025-02-14

**Authors:** Yun‐Ga Wu, Wei‐Lei Zhou, Yugui Qiu, Siwei Wang, Jinglin Liu, Yong Chen, Xiufang Xu, Yu Liu

**Affiliations:** ^1^ College of Chemistry and Material Science Inner Mongolia Key Laboratory of Chemistry for Nature Products and Synthesis for Functional Molecules Innovation Team of Optical Functional Molecular Devices Inner Mongolia Minzu University Tongliao 028000 P. R. China; ^2^ College of Chemistry State Key Laboratory of Elemento‐Organic Chemistry Nankai University Tianjin 300071 P. R. China; ^3^ College of Chemistry and Environmental Science Xinjiang Key Laboratory of Novel Functional Materials Chemistry Kashi University Kashi 844000 P. R. China

**Keywords:** 4D assembly, encryption and visual sensing, lanthanide supramolecular, multicolor phosphorescence, time‐dependent

## Abstract

Supramolecular dynamic room temperature phosphorescence (RTP) is the focus of current research because of its wide application in biological imaging and information anti‐counterfeiting. Herein, a time‐dependent supramolecular lanthanide phosphorescent 4D assembly material with multicolor luminescence including white, which is composed of 4‐(4‐bromophenyl)‐pyridine salt derivative (G), inorganic clay (LP)/Eu complex and pyridine dicarboxylic acid (DPA) is reported. Compared with the self‐assembled nanoparticle G, the lamellar assembly G/LP showed the double emission of fluorescence at 380 nm and phosphorescence at 516 nm over time. Within 60 min, the phosphorescence lifetime and the quantum yield increases from none to 7.4 ms and 27.53% respectively, achieving the time‐dependent phosphorescence emission, due to the limitation of progressive stacking of LP electrostatically driven “domino effect.” Furthermore, the 4D assembly of DPA and G/LP/Eu leads to a time‐resolved multicolor emission from colorless to purple to white, which is successfully applied to information multi‐level logic anti‐counterfeiting and efficiently antibiotic selective sensor.

## Graphical abstract

Supramolecular dynamic room temperature phosphorescence is the focus of current research because of its wide application in biological imaging and information anti‐counterfeiting. Herein, a time‐dependent supramolecular lanthanide phosphorescent 4D assembly material with multicolor luminescence including white for encryption and visual sensing, is reported which is composed of 4‐(4‐bromophenyl)‐pyridine salt h derivative, inorganic clay/Eu complex and pyridine dicarboxylic acid.

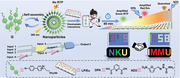

## 1 Introduction

Dynamic self‐assembly is common in various organisms in nature.^[^ ^1^ ^]^ In this process, weak non‐covalent interactions between molecules accurately produce effective and complex biological functions on demand, such as the formation of actin filaments, DNA transcription and replication, and other life activities.^[^ ^2^ ^]^ Inspired by this, imitating the ubiquitous dynamic self‐assembly to realize complex structures and functions can endow materials with intelligent bionic characteristics like self‐regulation, self‐healing, and self‐adaptation.^[^ ^3^ ^]^ Particularly, information security issues play a vital role in people's economy, health, and life in the current era of science and technology.^[^ ^4^ ^]^ Therefore, constructing dynamically tunable molecular luminescence systems through self‐assembly strategies, especially time‐dependent control, has brought new opportunities for the development of advanced data encryption materials.^[^ ^5^ ^]^ Tian et al.^[^ ^6^ ^]^ constructed a 4D dynamic multicolor fluorescent anti‐counterfeiting material using amphiphilic pyrene dyes, which modulated the time‐varying fluorescence transition from blue to orange during molecular assembly in tailored solvents. Yan et al.^[^ ^7^ ^]^ reported a transient programmable anti‐counterfeiting material based on cyclodextrin, in which the cyan fluorescence “lighting” time could be flexibly adjusted by controlling the arrangement of 4,4′‐biphenyldicarboxylic acid molecules through temperature and concentrations of cyclodextrin and Zn ions. Wang and Xiao et al.^[^ ^8^ ^]^ developed a time‐dependent single‐component fluorescent encryption system for the conversion of orange‐emitting nanoparticles to green‐emitting nanosheets in a CH_3_CN/H_2_O mixed solvent using a bridged cyanostyrene derivative.

On the other hand, organic room temperature phosphorescence (RTP) materials have witnessed rapid development in fields such as bioimaging,^[^ ^9^ ^]^ information encryption,^[^ ^10^ ^]^ and sensors^[^ ^11^ ^]^ in recent years due to their advantages of long lifetime and large Stokes shift. The supramolecular assembly strategy based on host‐guest interaction is convenient for preparation and avoids complex synthesis.^[^ ^12^ ^]^ More importantly, this method can easily adjust different luminescent properties of RTP materials by changing composition parameters and endowing them with fascinating dynamic characteristics, thus attracting extensive attention from researchers.^[^ ^13^ ^]^ Chi et al.^[^ ^14^ ^]^ doped commercial fluorescent dyes into the host molecular film of 4‐bromobenzophenone‐modified diphenyl phosphine oxide and achieved the dynamic RTP of guest molecules by controlling the concentration of residual oxygen in the matrix. Yang et al.^[^ ^15^ ^]^ designed and constructed an intelligent RTP material based on the hydrogen‐bonded organic framework host of trimesic‐acid/melamine and the phosphorescent guest of 4‐bromo‐1,8‐naphthalic anhydride, which is durable and effective in water. Ma et al.^[^ ^16^ ^]^ constructed a 3D phosphorescent Förster resonance energy transfer supramolecular organic framework with doped anionic dyes for the detection of drugs by assembling 4‐(4‐bromophenyl) pyridine‐modified tetraphenylmethane with cucurbit[8]uril. These RTP material design and fabrication strategies provide inspiring insights for information encryption and sensors. However, up‐to‐date, time‐dependent multicolor lanthanide supramolecular RTP materials in aqueous solution still face great challenges.

**Scheme 1:**
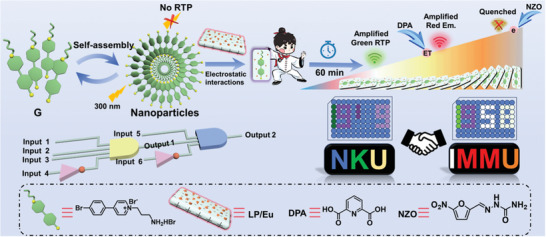
Schematic diagram of the construction of time‐dependent multicolor G/LP/Eu@DPA⊂NZO 4D supramolecular assembly.

Herein, we constructed a tunable time‐dependent supramolecular lanthanide phosphorescent 4D assembly material with multicolor emissions including white, which is composed of 4‐(4‐bromophenyl)‐pyridine salt derivative (G), inorganic clay (LP)/Eu complex and pyridine dicarboxylic acid (DPA). Interestingly, due to its unique negative surface and positive edge in aqueous solution,^[^ ^17^ ^]^ LP gradually forms a “domino effect” accumulation over time under electrostatic driving. This visually lights up double emission of fluorescence at 380 nm and phosphorescence at 516 nm over time. The phosphorescence lifetime and the quantum yield reach 7.4 ms and 27.53% in 60 min, respectively. Meanwhile, the fluorescence intensity at 380 nm continuously increases by 20 times, accompanied by a 5.5‐fold increase in quantum yield. Importantly, LP/Eu could emit pale red light with a quantum yield of 1.22% and a lifetime of 0.3 ms through the ion exchange of Eu^3+^ and Na^+^ on LP. After assembling with G, combined with the blue fluorescence and green phosphorescence of G, the dynamically adjustable purple‐to‐white light changes were achieved, demonstrating time‐dependent multicolor emissions including white. At the same time, the 4D assembly of DPA (anthracnose‐specific biomarker) and G/LP/Eu achieves rapid ratio enhancement (the luminescence intensity at 616 nm increases by 7 times, and the partial quenching efficiency of blue and green light reaches > 60%), accompanied by color changes from white to orange. Further, the secondary assembly G/LP/Eu@DPA can selectively detect antibiotics nitrofurazone (NZO), metronidazole (MAZ), and sulfamethazine (SAZ). The quenching efficiency of NZO (70 nM) at 616 nm is > 70%, while MAZ and SAZ are both below 30%. This dynamic multicolor and long‐lived assembly of “two birds with one stone” not only demonstrates multi‐level logical encryption of time‐dependent information but also enables selective detection of antibiotic furan (**Scheme** **1**).

## 2 Results and Discussion

**Figure 1:**
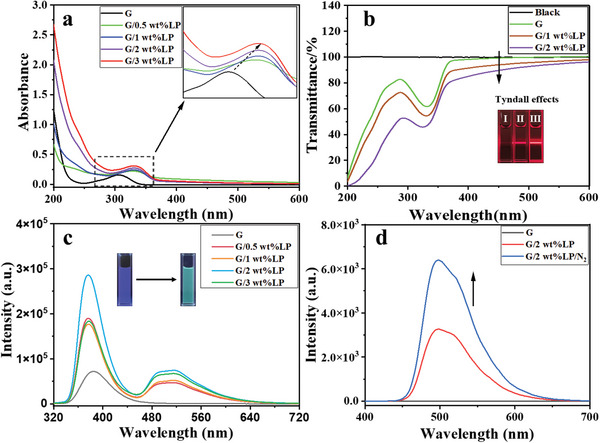
a) Absorption spectra of adding LP (from 0 to 3 wt.%) to G (0.01 mm) in water at 298 K; b) The optical transmittance at 450 nm with the different addition LP into G at 298 K. Inset: Tyndall effect for G with different additions of LP (I) 0 wt.%, (II) 1 wt.% and (III) 2 wt.%; c) The prompt photoluminescence spectra of G with LP (from 0 to 3 wt.%) in water at 298 K (λ_ex_ = 300 nm); d) Phosphorescence emission spectra (delay 0.1s) of G/LP in water before and after N_2_ bubbling at 298 K ([G] = 0.01 mm, LP = 2wt.%, λ_ex_ = 300 nm).

3‐Bromopropane‐1‐amine hydrobromic acid was added to the solution of 4‐(4‐bromophenyl) pyridine in acetonitrile. After refluxing and heating for 2 h, the solution was cooled to room temperature and filtered to obtain a yellow product 4‐(4‐bromophenyl)‐pyridin‐1‐ium (G) with a yield of 67% (Figures S1–S4, Supporting Information). The positive charge of G makes it suitable for assembling with LP with an orthogonally charged surface (negative charge) and edge (positive charge). The optical properties of the aqueous solution of G with LP were studied by UV‐visible spectroscopy and photoluminescence spectroscopy. The UV‐visible spectrum of G showed that the maximum absorption intensity of G at 307 nm gradually increased with the addition of LP, accompanied by a red shift of the maximum to 330 nm (**Figure** **1**a). To further understand the assembly process after the addition of LP in G, the change in optical transmittance was also monitored. As shown in Figure 1b, with pure water as the reference sample, G did not show any change in optical transmittance. However, the addition of 1–2% LP in G led to a sharp decrease in the optical transmittance of G/LP at 450 nm, indicating that the electrostatic interaction between them was conducive to the formation of large nanostructures. Moreover, the assembly G/LP and monomer G could also be easily distinguished by the Tyndall effect. After the addition of LP to G, the electrostatic interaction between LP and G, as well as the hydrogen bond and π‐π stacking interaction between G molecules, may change the electron density of G molecules and thus change the excited state properties of G molecules, thereby enhancing their absorption at specific wavelengths. Then, the formation of the G/LP assembly changes the electronic structure of the G molecule and will cause a change in its luminescent properties. Through photoluminescence spectroscopy (Figure 1c), we found that when adding LP at concentrations of 0.5, 1, and 2 wt.% into G, the fluorescence emissions of G at 380 nm were continuously enhanced. The luminescence intensity increased by 4 times and the quantum yield was enhanced by 1.3 times, while the lifetimes remained almost unchanged (1ns and 1.3 ns, respectively). At the same time, an obvious new phosphorescence emission peak appears at 516 nm in combination with delayed phosphorescence spectra. When LP is added to 2wt.%, the phosphorescence quantum yield reaches 7.93% and the lifetime is 2 ms. After introducing N_2_ gas, the luminescence intensity increased by 2 times, the lifetime increased by 1.8 times, and the yield increased by 2.4 times, further proving the phosphorescence at 516 nm, while after introducing N₂ into the G monomer molecules, the delayed phosphorescence spectrum remained unchanged, highlighting that the addition of LP caused changes in the optical properties of the G molecules (Figure 1d; Figures S5–S8, Supporting Information). However, when LP is added to 3 wt.%, both the fluorescence at 380 nm and the phosphorescence at 516 nm begin to quench, which may be caused by the competitive interaction of excess charge. In the controlled experiments (Figure S9a–d, Supporting Information), the quaternary ammonium cationic *β*‐cyclodextrin (ACD) and ethylene imide polymer (PEI) were added to LP solution to neutralize the negative charge on LP and then assembled with G. We found that the luminescence of the assembly LP/ACD or LP/PEI is greatly quenched after further assembly with G, which is mainly due to the negative charge on LP is neutralized by the positive charge of ACD or PEI. On the other hand, we continued to introduce negatively charged sulfobutylether‐*β*‐cyclodextrin (SBE‐*β*‐CD) into the assemblies LP/ACD/G or LP/PEI/G and found that with the increase of the amount of SBE‐*β*‐CD, the luminescence of the assemblies LP/ACD/SBE‐*β*‐CD/G or LP/PEI/SBE‐*β*‐CD/G continuously enhanced. In addition, when a rigid macrocyclic host cucurbit[8]uril (CB ^[^ ^8^ ^]^) was added to the G aqueous solution, a new phosphorescence emission peak at 516 nm appeared, but the intensity of the emission peak was much smaller than that of the assembly LP/G (Figure S10a–e, Supporting Information). In addition, the negatively charged sodium carboxymethyl cellulose (CMC) and hyaluronic acid (HA) were added to the aqueous solution of G, and there was no obvious emission peak at 516 nm (Figure S11a–d, Supporting Information). This phenomenon proves that the non‐covalent interaction between LP and G is mainly electrostatic interaction and the stronger rigid structure of LP is in favor of limiting the non‐radiative transition of G molecules.

**Figure 2:**
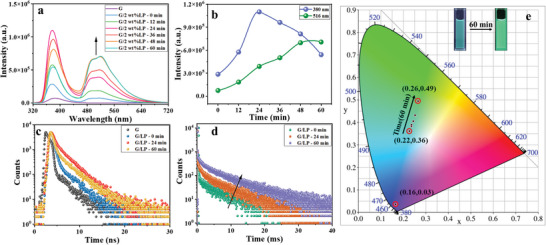
a) The dynamic photoluminescence spectra of G with LP in water at 298 K (λ_ex_ = 300 nm); b) The dynamic photoluminescence emission intensity changes diagram of G with LP in water at 298 K (λ_ex_ = 300 nm); c) The fluorescence dynamic lifetime decay curves of G/LP at 380 nm at 298 K; d) The phosphorescence dynamic lifetime decay curves of G/LP at 516 nm at 298 K; e) The corresponding CIE chromaticity graph of the dynamic changed spectra of G with LP in water at 298 K (λ_ex_ = 300 nm).

Interestingly, we accidentally discovered that the luminescence properties of the assembly G/LP formed after adding LP to G are dynamically time‐dependent. Taking the 2wt.% LP with the best photoluminescence properties as an example, the change of its photoluminescence properties was monitored after ultrasound for 60 min. The enhanced fluorescence at 380 nm after adding LP increased with time. The fluorescence emission intensity at 380 nm reached the best state at 24 min and then stabilized at 60 min. The luminescence intensity increased by 4 times, accompanied by a two‐fold increase in quantum yield (from 7.32% to 14.85%) with a steady fluorescence lifetime (from 1.3 to 2 ns). Moreover, the new phosphorescence at 516 nm also changed significantly. After 60 min, the luminescence intensity increased by 9.5 times, and the phosphorescence quantum yield reached 27.53%, i.e., increasing by 3.4 times. In addition, the phosphorescence lifetime increased to 7.4 ms, i.e., an increase of 3.7 times (**Figure** **2**a–d; Figures S11–S15, Supporting Information). On a 2D projection of the CIE (Commission Internationale de l'Eclairage) xy chromaticity diagram (Figure 2e), we can also observe the changing trend of the luminous color after adding LP to G for 60 min, from cyan (0.22, 0.36) to bright green (0.26, 0.49). Additionally, G/LP assemblies exhibited satisfactory photostability, and their spectra hardly changed after the natural light irradiation for 72 h, and the delayed phosphorescence spectra of G/LP under natural light, dark conditions, salt, and acid conditions did not change. Furthermore, G/LP exhibits excellent repeatability and stability in both natural light and salt environments (Figure S16a–d, Supporting Information), which is an important indicator of the luminescent materials. Subsequently, to explore the assembly mode, the changes of zeta potential and morphology were studied after the formation of the assembly. The potential of G was measured to be 7.64 mV, showing a positive charge that can be assembled with a negatively charged host compound. LP is inorganic rigid silicate natural clay with orthogonal charge. Once LP (zeta potential of −50.34 mV) forms an assembly with G, the zeta potential increases to −38.42 mV, indicating that electrostatic interaction occurs between positively charged G and the negatively charged surface of LP (Figure S17, Supporting Information). The morphology changes were observed by transmission electron microscopy (TEM). The TEM images of the assembly G/LP showed many lamellar aggregates, which were very different from the spherical morphology of G, revealing the formation of the assembly (Figure S18, Supporting Information).

**Figure 3:**
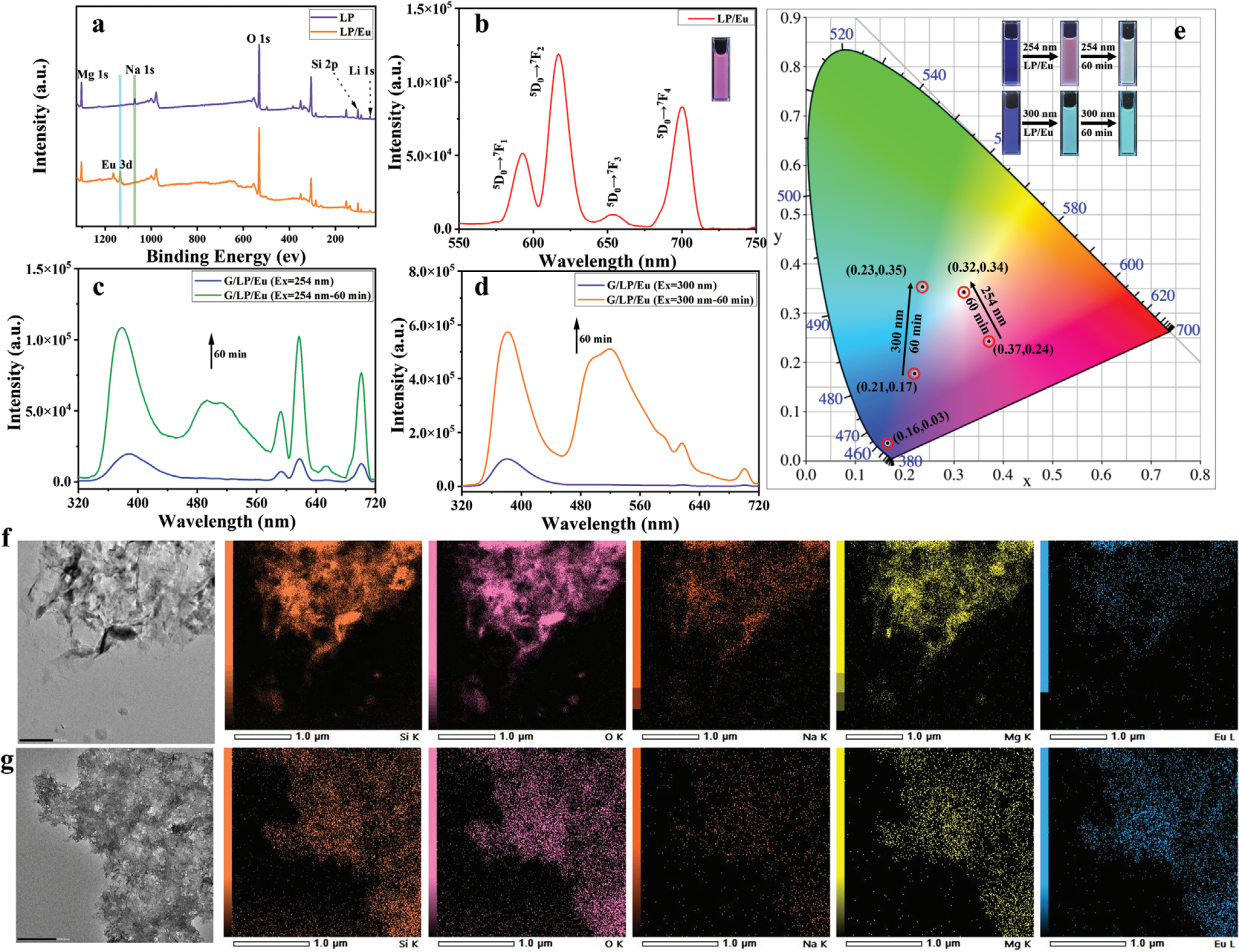
a) X‐ray photoelectron spectra (XPS) of LP and LP/Eu; b) Fluorescence spectra of LP/Eu in water at 298 K (λ_ex_ = 254 nm); c)The dynamic prompt photoluminescence spectra of G/LP/Eu in water at 298 K (λ_ex_ = 254 nm, LP = 0.3 wt.%); d) The dynamic prompt photoluminescence spectra of G/LP/Eu in water at 298 K (λ_ex_ = 300 nm, LP = 0.3 wt.%); e) The Corresponding CIE chromaticity graph of the dynamic changed spectra of G with LP/Eu under different excitation in water at 298 K (λ_ex_ = 254 nm and λ_ex_ = 300 nm, LP = 0.3 wt.%); Transmission electron microscopy (TEM) and corresponding mapping images of f) LP and g) LP/Eu.

In addition, multi‐color luminescence, especially white light with high color fidelity and low distortion,^[^ ^18^ ^]^ is widely utilized in lighting and anti‐counterfeiting applications. To obtain white light emission, we prepared the LP/Eu host complex by conducting a simple ion exchange reaction to replace sodium ions with lanthanide europium ions in LP.^[^ ^19^ ^]^ The composition and chemical bonds of LP and LP/Eu were investigated by X‐ray photoelectron spectrometer (XPS) (**Figure** **3**a). The full‐spectrum peaks of LP were at 49.72, 101.72, 531.72, 1071.73, and 1303.73 eV, corresponding to Li(1s), Si(2p), O(1s), Na(1s), and Mg(1s), respectively. In contrast, the Na 1s peak vanished, and the Eu 3d peak emerged in the XPS spectrum of the LP/Eu complex, indicating the successful preparation of the LP/Eu complex. Further, the TEM images of LP and LP/Eu both reveal a large number of layered aggregates (Figure 3f), and the TEM mapping images show that compared with LP, the content of Na^+^ in LP/Eu is reduced, while the content of Eu^3+^ is significantly increased (Figure 3g). These all prove that the exchange of LP/Eu is successful. The fluorescence emission spectra of the LP/Eu complexes were measured with 254 nm excitation as shown in Figure 3b, where characteristic emission peaks of europium at 593 nm (^5^D_0_→^7^F_1_), 616 nm (^5^D_0_→^7^F_2_), 653 nm (^5^D_0_→^7^F_3_), and 701 nm (^5^D_0_→^7^F_4_) were observed.^[^ ^20^ ^]^ The fluorescence lifetime and quantum yield were 0.3 ms and 1.22%, respectively (Figures S19–S21, Supporting Information). Subsequently, the red luminescent LP/Eu complex was used as the host to replace the above‐mentioned LP and added to the G aqueous solution. The blue fluorescence at 380 nm and the green phosphorescence at 516 nm increased with the addition of 0.3 wt.% LP/Eu, demonstrating the same phenomenon in the assembly of G/LP/Eu. Meanwhile, we also observed the characteristic emission peak of rare‐earth europium, indicating that the ion‐exchanged host LP/Eu does not affect the luminescence of G. Additionally, with 60 min of ultrasonic dissolution, the luminescence at 380 and 516 nm is further enhanced. Significantly, due to the blue fluorescence, green phosphorescence, and red rare‐earth luminescence upon adding LP/Eu into the guest G, the luminescence of the assembly under 254 nm excitation changes from blue to light pink and gradually becomes a strong white light emission after 60 min (Figure 3c–e), with the corresponding CIE coordinates being (0.16, 0.03), (0.37, 0.24), and (0.32, 0.34), respectively. Under the excitation wavelength of 300 nm, the assembly G/LP/Eu emits faint blue‐green light due to the inability to excite the luminescence of rare‐earth europium and can emit green light after 60 min, corresponding to the CIE coordinates of (0.23, 0.35) and (0.21, 0.17), respectively (Figure 3d,e). The addition of excess LP/Eu in the G aqueous solution can emit strong pink light at a 254 nm excitation wavelength, and light‐yellow light can be emitted after 60 min, with CIE coordinates of (0.44, 0.32) and (0.39, 0.40), respectively. At the same time, corresponding green light and bright green light are emitted at a 300 nm excitation wavelength, with CIE coordinates of (0.25, 0.34) and (0.24, 0.40), respectively (Figures S22, S23a–c, Supporting Information). Then, multi‐color full‐spectrum luminescence, including white light, can be achieved by adjusting the time and the ratio of the guest G and the host LP/Eu, which is conducive to the construction of advanced smart materials.

**Figure 4:**
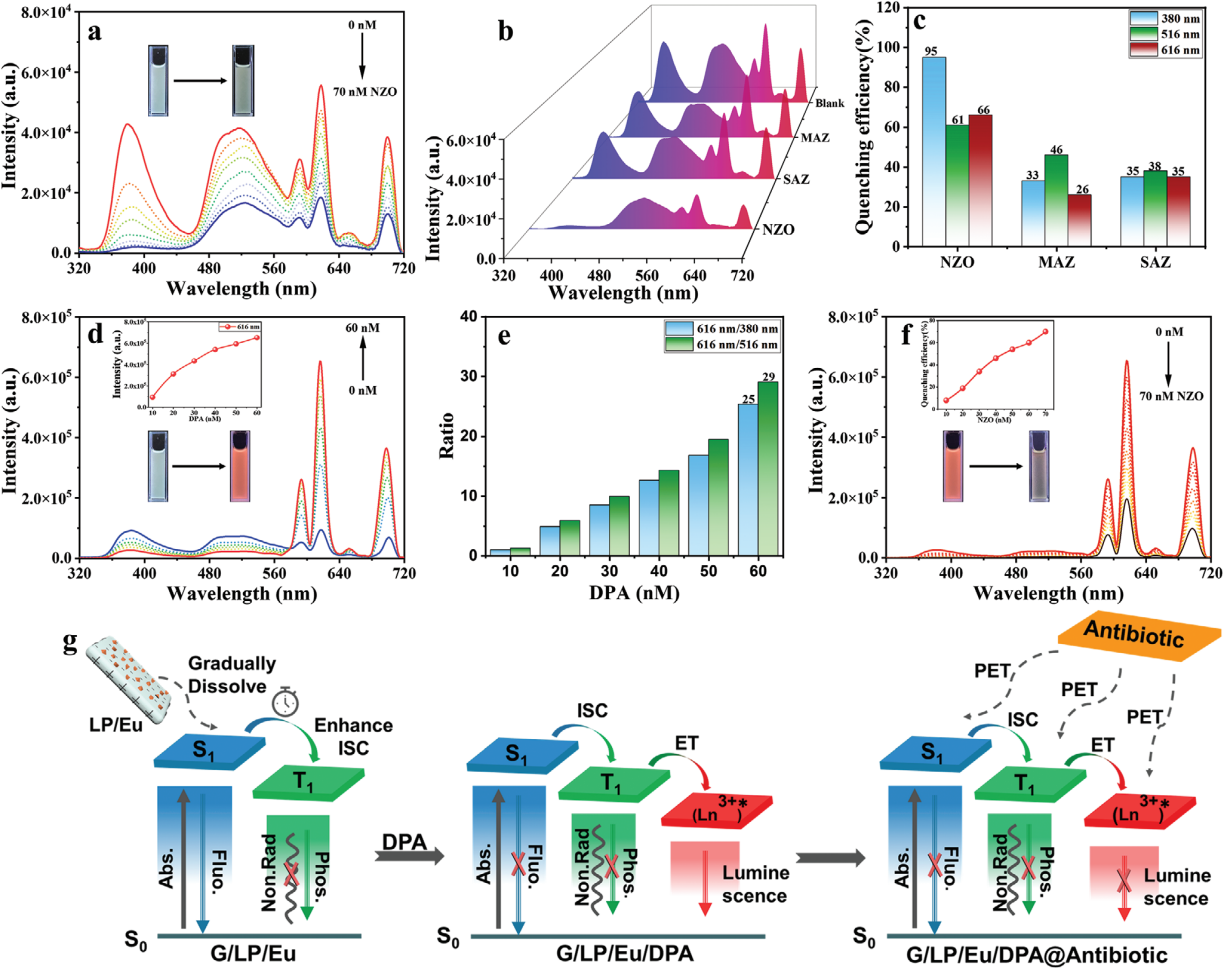
a) The prompt photoluminescence spectra of aqueous solution G/LP/Eu by adding NZO at 298 K (λ_ex_ = 254 nm); b) Emission spectra of G/LP/Eu for different antibiotics in aqueous solution (λ_ex_ = 254 nm); c) The luminescent quenching efficiency for the emission of the G/LP/Eu with the different antibiotics at 380, 516, and 616 nm; d) The prompt photoluminescence spectra of aqueous solution G/LP/Eu by adding DPA at 298 K (λ_ex_ = 254 nm); e) the ratio of 616 nm/380 nm and 616 nm/516 nm values of G/LP/Eu under 254 and 300 nm irradiation; f) The prompt photoluminescence spectra of DPA followed by NZO added in G/LP/Eu in water at 298 K (λ_ex_ = 254 nm); g) The graphical possible working mechanism of phosphorescence energy transfer process and sensing.

On the other hand, the G/LP/Eu assemblies can be applied to the detection of various antibiotics in water due to their excellent dynamic lanthanide phosphorescence properties and good water solubility. As shown in Figure S24a–c (Supporting Information), different antibiotics (70 nm), such as nitrofurazone (NZO), nitrofurazone (MAZ), and sulfamethazine (SAZ), were added to the G/LP aqueous solution. The quenching efficiency at 380 and 516 nm was calculated, and NZO has a higher quenching efficiency (>95% and >70%, respectively) than MAZ (<70%) and SAZ (<70%) (Figure S24d–f, Supporting Information). When adding three antibiotics to LP/Eu to calculate the quenching efficiency, NZO (the quenching efficiency at 616 nm is 70%) also has better quenching efficiency than others (MAZ <20% and SAZ <30%) (Figure S25, Supporting Information). Then, the white light G/LP/Eu assembly has a better selective detection effect on NZO, as shown in **Figure** **4**a–c. The quenching efficiency of NZO at 380, 516, and 616 nm in G/LP/Eu reached 95%, 61%, and 66%, respectively (Figure S26, Supporting Information), while the quenching efficiency of MAZ and SAZ is relatively weak (<50%) (Figure S27, Supporting Information).

More importantly, bacillus anthracis is extremely fatal. Inhalation of only 10^3^–10^4^ bacterial spores can lead to death, and the mortality rate is as high as 25%.^[^ ^21^ ^]^ Thus, the detection of this pathogen is particularly crucial. The calcium salt of pyridine dicarboxylic acid (DPA) accounts for 5–15% of bacterial dry weight and is a specific biomarker of anthrax. The luminescence of LP/Eu/DPA after secondary complexation is gradually enhanced (from pink to bright red) with the gradual addition of DPA to 70 nM in the LP/Eu complex, in which the luminescence intensity is increased by 234 times, the yield is increased by 4 times, and the lifetime is increased from 0.3 to 0.4 ms (Figures S28‐S31, Supporting Information). By gradually adding 70 nM DPA to the dynamic assembly LP/G, we found that the luminescence at 380 and 516 nm was gradually quenched with quenching rates of 36% and 42% (Figure S32, Supporting Information), respectively. So, we believe that the white‐light‐emitting assembly G/LP/Eu is beneficial to the secondary assembly with DPA to achieve proportional high‐selectivity detection. Then, after DPA was gradually added to the G/LP/Eu assembly for 4D assembly, the characteristic emission peaks of Eu^3+^ in the assembly (593, 616, 653, and 701 nm) increased sharply (the luminous intensity increased by seven times). The emission intensity of Eu^3+^ at 616 nm was doubled, while the fluorescence at 380 nm and the phosphorescence emission at 516 nm were weakened, and the quenching efficiency was surprisingly achieved at 70% and 68%, respectively (Figure 4d,e; Figure S33, Supporting Information), accompanied by the transition from white light to orange‐red light. Thus, even in the slightest risk of anthrax, the G/LP/Eu assembly can be efficiently used for proportional detection. Importantly, we can further achieve the selective detection of NZO in aqueous solutions using the 4D assembly G/LP/Eu@DPA. The quenching efficiency at 616 nm can reach 70% (Figure 4f), and with the further quenching of blue fluorescence and green phosphorescence, the orange luminescence of the solution becomes almost colorless. To investigate the effects of the electrostatic interactions between the negatively charged LP and the positively charged guest molecule G on the phosphorescence intensity of molecule G, we calculated the energy difference between the first excited singlet state (S_1_) and the first excited triplet state (T_1_) of molecule G in the presence of an applied background charge. It is worth noting that, due to the complexity of the LP structure, it is often challenging to achieve convergence in direct calculations involving the guest molecule G in the presence of LP. Therefore, we adopted a method that involves directly introducing a background negative charge instead of the LP around the guest molecule G to model the studied system. Moreover, in the calculation, we set 0, 0.25, 0.5, 0.75, and 1 units of negative charge, respectively, around the guest molecule G to disclose the effect law of the amount of the negative charge around the guest molecule G on the phosphorescence intensity. According to the calculation results shown in (Figure S34a, Supporting Information), when the background negative charge around the molecule is 0, the energy gap between the molecule's first singlet excited state (S_1_) and the first triplet excited state (T_1_) is 0.9708 eV. When the background negative charge around the molecule is −1.0, this energy gap decreases to 0.951 eV. This indicates that the presence of negative charges around the molecule promotes intersystem crossing from the S_1_ excited state to the T_1_ excited state, thereby enhancing the phosphorescence intensity of the molecule. Furthermore, as shown in (Figure S34b, Supporting Information), there is a clear relationship between the S_1_‐T_1_ energy gap and the quantity of background charge, with the energy gap decreasing as the negative charge increases. This further indicates that the presence of negative charges around the molecule should be conducive to the generation of phosphorescence. In order to investigate the influence of external charges on the electronic structure of molecule G, we explored the charge distribution of key atoms within molecule G in the presence of different quantities of background charge. As shown in the electrostatic potential map in (Figure S34c, Supporting Information), the pyridine ring moiety of the molecule is positively charged (blue area), while the bromobenzene and amino groups at both ends of the molecule are negatively charged (green area). Further analysis of the charge quantities distributed on the bromine atom and nitrogen atom at both ends of the molecule revealed that as the quantity of the background negative charge increases, the electrons in the positively charged part (i.e., the pyridine ring moiety) of the molecule gradually transfer to the negatively charged ends. Moreover, as the quantity of the background negative charge increases, the energy of molecule G decreases, thus molecule G becoming more stable, as shown in (Figure S34d, Supporting Information). At the same time, LP gradually dissolves and the electrostatically driven “House of Cards” between LPs gradually assembles with G over time to form a limiting effect of “domino effect” stacking, as well as reducing the collision with oxygen in water to a certain extent (Figure 4g). The addition of DPA forms a coordination effect with rare‐earth ions, and energy transfer leads to the enhancement of luminescence. The fluorescence (380 nm) and phosphorescence (516 nm) quenching mechanism of DPA may be caused by photoinduced electron transfer (PET) and co‐absorption with the excited state of G/LP/Eu, as well as interaction with rare‐earth ions energy transfer. Similarly, the quenching of fluorescence, phosphorescence, and rare earth ion luminescence is due to PET and co‐absorption caused by the addition of antibiotics.

**Figure 5:**
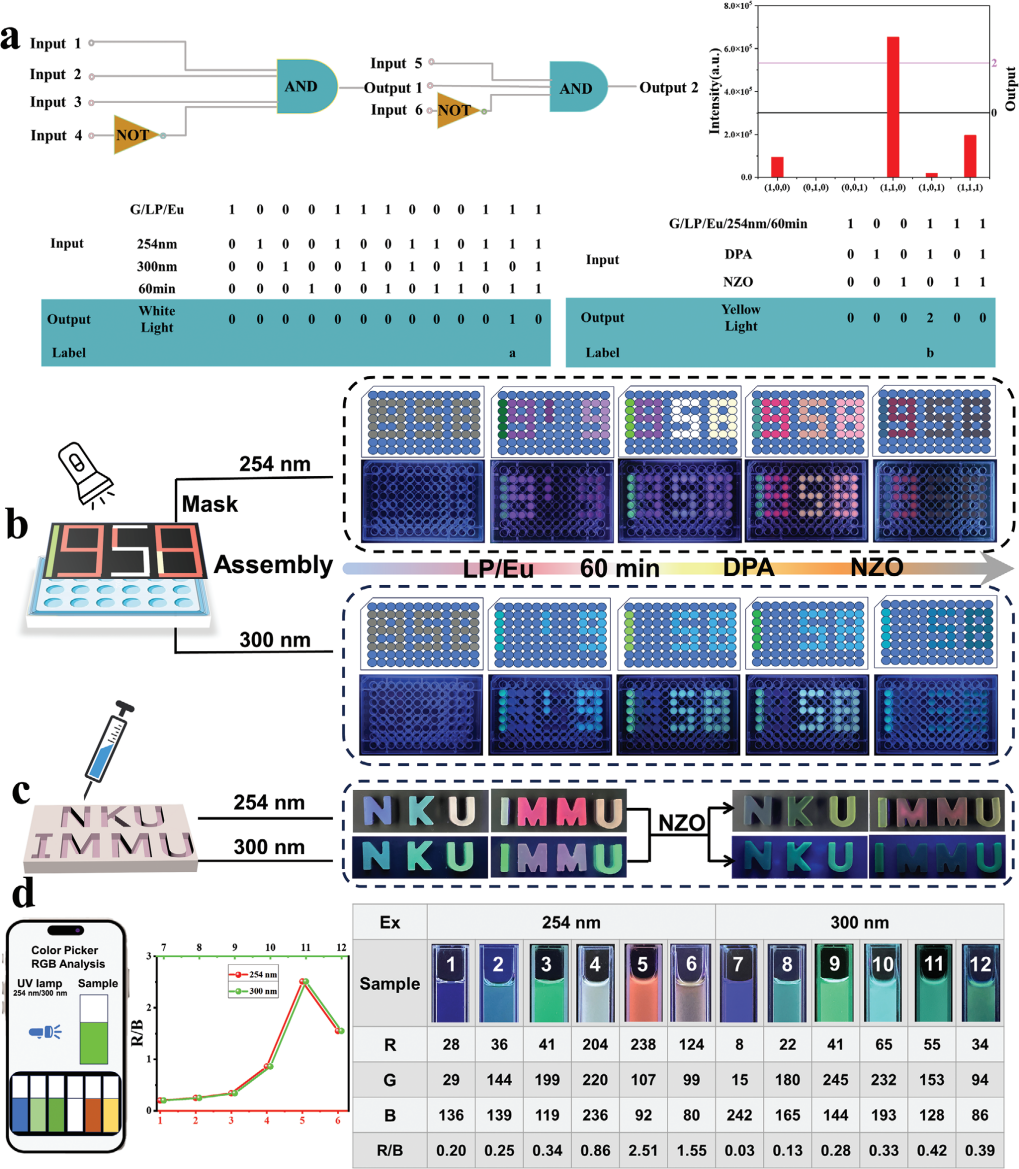
a) Combinational logic gate systems based on luminescent colors of assemblies and the corresponding truth tables(a and b represent the white and yellow light outputs of the logic gate respectively); b) Photographs of multilevel information storage with supramolecular ink under 254 and 300 nm irradiation ([G] = 0.01 mm, [LP] = 2 wt.%, DPA = 60 nm, NZO = 70 nm); c) Schematic diagram and photos of letters “NKU” and “IMMU” gel testing NZO([G] = 0.01 mm, [LP] = 2 wt.%, DPA = 60 nm, NZO = 70 nM); d) The numbers “1‐6” and “7‐12” represent the R, G, B, and R/B values of the photos of “G, G/LP‐0 min, G/LP‐60 min, G/LP/Eu, G/LP/Eu/DPA, G/LP/Eu/DPA/NZO” under 254 and 300 nm irradiation, respectively.

This dynamic time‐dependent “domino effect” tunable multicolor phosphorescent 4D assembly can be applied to the multi‐layer logic gate anti‐counterfeiting system. The device defines different components of the component as input, and the white light emission with a 254 nm excitation is defined as output (**Figure** **5**a). According to the white light output recorded as “1” and the non‐white light output recorded as “0,” a suppressed continuous logic gate system with light output is designed. It is worth noting that the output “0” and “1” states of the current logic gate represent several different types of supramolecular assembly. In the logic gate system, when G/LP/Eu/254 nm coexists, the white light emission can be “locked”, and 300 nm is used as a non‐gate to “mute” the output signal, and the output white light is then assembled differently with DPA and NZO. This continuous logic‐gate system defines the fluorescence emission intensity at 616 nm with a 254 nm excitation as output. The fluorescence intensity of the output above 5 × 10^5^ is recorded as “2,”, and the fluorescence intensity below 5 × 10^5^ is recorded as “0”. When G/LP/Eu/254 nm/DPA coexists, yellow light emission can be “locked”, and NZO is used as a non‐gate to “mute” the output signal, which is clearly shown in the truth table. This simple logic gate based on LP supramolecular assembly can output complex information after adding multiple input components, which provides great potential for the construction of complex logic gate circuits. In addition, this LP supramolecular assembly is suitable for multi‐level information encryption. On the porous plate (Figure 5b), the host LP was added into G, LP/Eu was added into the aqueous solution, and G, as well as excess LP/Eu adding into G, the luminescent characters “1919” in dark green, red, light red and lavender could be presented under 254 nm irradiation, respectively. The G/LP/Eu assembly was added to the small “1,” and after 60 min the “1958” characters were given glowing in different colors (bright green, red, white, and light yellow, respectively). Further adding DPA, the “1958” could be changed into light green, bright red, yellow, and orange light, respectively. Finally, almost all quenched after adding NZO, only 9 can faintly show a weak red. The porous plate of each step can emit different color luminescent characters “119” under 300 nm irradiation, which are light green “1” and light blue “1” and “9,” respectively. In one hour of placement, the number showed a brighter “119” which was progressively quenched with the continuous addition of DPA and NZO. As shown in Figure 5c, we used agarose to further form a modular gel of aqueous solutions of different assembly G, G/LP, LP/Eu, G/LP/Eu, LP/Eu@DPA and G/LP/Eu@DPA to spell out “NKU” and “IMMU.” Under 254 nm irradiation, the letters “NKU” of different colors were blue “N” (G), light green “K” (G/LP) and light yellow “U” (LP/Eu), respectively and “IMMU” was white “I” (G/LP/Eu@1 h), bright red “M” (G/LP/Eu@1h⊂DPA)and orange “U” (LP/Eu⊂DPA), respectively. Under the irradiation of 300 nm, the letters “NKU” with different colors were light green “N”, bright green “K” and “U”, respectively, and “IMMU” was bright green “I,” colorless “M” and bright green “U,” respectively, writing NKU@IU. After the antibiotic NZO was added, the luminescence was almost completely quenched at either 254 or 300 nm, but it still gave different weak colors. Finally, a smartphone was used to collect a series of pictures excited by 254 and 300 nm and analyze them by color recognition software to obtain their RGB values. By calculating the R/G values, a good linear relationship was obtained with the addition of LP, after 60 min and with the addition of DPA, and decreased sharply after the addition of NZO (Figure 5d).

## 3 Conclusion

In summary, an advanced logic‐gate anti‐counterfeiting material with time‐mode and excitation wavelength encryption was successfully developed by utilizing a dynamic multicolor lanthanide phosphorescent 4D supramolecular assembly based on inorganic rigid silicate natural LP with orthogonal charge, phosphorescent molecule G, and Eu ion. In an aqueous solution, due to the electrostatically driven “domino effect” dynamic stacking of LP, the time‐dependent green phosphorescence at 516 nm of G is activated, with a lifetime of 7.4 ms and a quantum yield of 27.53%. At the same time, the blue fluorescence at 380 nm was lit up with the continuous increase in luminescence intensity by 15 times, and the quantum yield by 2 times. Interestingly, LP/Eu obtained through ion exchange is assembled with G, and a 4D assembly with DPA is also conducted to obtain a time‐dependent multicolor phosphorescent character conversion from “NKU@1919” to “IMMU@1958.” Additionally, the multicolor dynamic assembly shows highly sensitive and selective detection of antibiotics. This study not only paves a new way for the construction of advanced information anti‐counterfeiting but also provides a simple and feasible method for the specific detection of DPA and antibiotics, possessing great potential application value in supramolecular intelligent materials and therapeutic research.

## Conflict of Interest

The authors declare no conflict of interest.

## Supporting information



Supporting Information

## Data Availability

The data that support the findings of this study are available from the corresponding author upon reasonable request.
