# Supplementary material for: 4D Assembly of Time‐dependent Lanthanide Supramolecular Multicolor Phosphorescence for Encryption and Visual Sensing
Source: Adv Sci (Weinh). 2025 Feb 14;12(14):2415418. doi: 10.1002/advs.202415418 (PMC11984880; doi:10.1002/advs.202415418)
Supplement: Supplementary file 1 — Supporting Information [file ADVS-12-2415418-s001.doc]

Supporting Information

**Four-dimensional Assembly of Time-dependent Lanthanide Supramolecular Multicolor Phosphorescence for Encryption and Visual Sensing**

*Yun-Ga Wu^+^,^[a]^ Wei-Lei Zhou^+^*,^[a], [b]^ Yugui Qiu,^[b], [c]^ Siwei Wang,^[a]^ Jinglin Liu,^[a]^ Yong Chen,^[b]^ Xiufang Xu,^[b]^ Yu Liu*^[b]^*

[a] Y.-G. Wu, W.-L. Zhou, S. Wang, J. Liu

College of Chemistry and Material Science, Inner Mongolia Key Laboratory of Chemistry for Nature Products and Synthesis for Functional Molecules, Innovation Team of Optical Functional Molecular Devices, Inner Mongolia Minzu University, Tongliao 028000, P. R. China.

[b] W.-L. Zhou, Y. Qiu, Prof. Y. Chen, Prof. X. Xu, Prof. Y. Liu

College of Chemistry, State Key Laboratory of Elemento-Organic Chemistry, Nankai University, Tianjin 300071, P. R. China.

[c] Y. Qiu

College of Chemistry and Environmental Science, Xinjiang Key Laboratory of Novel Functional Materials Chemistry, Kashi University, Kashi 844000, China.

E-mail: zhouweilei@imun.edu.cn; yuliu@nankai.edu.cn

[+] These authors contributed equally to this work.

**Table of Contents**

**I. Experimental section**

**II. Figures**

**Figure S1. The Synthetic route of G.**

**Figure S2. ^1^H NMR (500 MHz, D_2_O, 298 K) spectrum of compound G.**

**Figure S3. ^13^C NMR (125 MHz, D_2_O, 298 K) spectrum of compound G.**

**Figure S4. ESI mass spectrum of G.**

**Figure S5. The Phosphorescence lifetime decay curves of G/LP/N_2_ in aqueous solution.**

**Figure S6. The phosphorescence lifetime decay fitting curves of G/LP/N_2_ in aqueous solution.**

**Figure S7. The phosphorescence quantum yields of G/LP/N_2_ in aqueous solution.**

**Figure S8. Phosphorescence emission spectra of G in water before and after N2 bubbling at 298 K.**

**Figure S9. (a) Fluorescence spectra and (b) phosphorescence spectra of LP/ACD/G, (c) Fluorescence spectra and (d) phosphorescence spectra of LP/PEI/G.**

**Figure S10. (a) Fluorescence spectra and (b) phosphorescence spectra of LP/ACD/SBE-*β*-CD/G, (c) Fluorescence spectra and (d) phosphorescence spectra of LP/PEI/ SBE-*β*-CD/G, (e) Phosphorescence emission spectra of G/CB[8] in water before and after N_2_ bubbling at 298 K.**

**Figure S11. (a) Fluorescence spectra and (b) phosphorescence spectra of G/CMC, (c) Fluorescence spectra and (d) phosphorescence spectra of G/HA.**

**Figure S12. The fluorescence lifetime decay fitting curves of(a) G, (b) G/LP-0 min, (c) G/LP-24 min and (d) G/LP-60 min in aqueous solution.**

**Figure S13. The phosphorescence lifetime decay fitting curves of (a) G/LP-0** **min, (b) G/LP-24 min and (c) G/LP-60 min in aqueous solution.**

**Figure S14. The fluorescence quantum yields of (a)G, (b) G/LP-0 min, (c) G/LP-24 min and (d) G/LP-60 min in aqueous solution.**

**Figure S15. The phosphorescence quantum yields of (a) G/LP-0 min, (b) G/LP-24 min and (c) G/LP-60 min in aqueous solution.**

**Figure S16. Phosphorescence emission spectra of (a) G/LP assembly solution under natural light for 72 hours at 298 K, (b) The phosphorescence emission spectra of the G/LP assembly solution at 298 K under different conditions, (c) The dynamic Phosphorescence emission spectra of G with LP in water at 298 K, (d) The dynamic Phosphorescence emission spectra of G/LP/NaCl in water at 298 K.**

**Figure S17. Zeta potential of (a) LP, (b) G and (c) G/LP in aqueous solution at 298 K.**

**Figure S18. Transmission electron microscopy (TEM) images of (a) G and (b) G/LP.**

**Figure S19. The fluorescence lifetime decay curves of LP/Eu in aqueous solution.**

**Figure S20. The fluorescence lifetime decay fitting curves of LP/Eu in aqueous solution.**

**Figure S21. The fluorescence quantum yields of LP/Eu in aqueous solution (The value ranges from 570 nm to 750 nm, λ_ex_ = 300 nm).**

**Figure S22. The corresponding CIE chromaticity graph of the dynamic changed spectra of G with the excess LP/Eu in water at 298 K (λ_ex_ = 300 nm).**

**Figure S23. (a) The prompt photoluminescence spectra of G aqueous solution; (b) The dynamic prompt photoluminescence spectra of G aqueous solution with excess LP/Eu under 254 nm excitation; (c) The dynamic prompt photoluminescence spectra of G aqueous solution with excess LP/Eu under 300 nm excitation.**

**Figure S24. The prompt photoluminescence spectra of (a) NZO, (b) MAZ, and (c) SAZ added to G aqueous solution; The luminescence quenching efficiencies of (d) NZO, (e) MAZ, and (f) SAZ corresponding to G/LP (λ_em_ = 380 nm and λ_em_ = 516 nm).**

**Figure S25. The prompt photoluminescence spectra of (a) NZO, (b) MAZ, and (c) SAZ added to LP/Eu aqueous solution; The luminescence quenching efficiencies of (d) NZO, (e) MAZ, and (f) SAZ corresponding to LP/Eu (λ_em_ = 616 nm).**

**Figure S26. The luminescence quenching efficiencies of NZO corresponding to G/LP.**

**Figure S27. The prompt photoluminescence spectra of (a) MAZ, and (b) SAZ added to G/LP/Eu aqueous solution; The luminescence quenching efficiencies of (c) MAZ and (d) SAZ corresponding to G/LP/Eu (λ_em_ = 380 nm, λ_em_ = 516 nm and λ_em_ = 616 nm).**

**Figure S28. (a) The prompt photoluminescence spectra of (a) DPA added to LP/Eu aqueous solution. (b) DPA enhances the emission intensity curve of LP/Eu.**

**Figure S29. The fluorescence lifetime decay curves of LP/Eu/DPA in aqueous solution.**

**Figure S30. The fluorescence lifetime decay fitting curves of LP/Eu/DPA in aqueous solution.**

**Figure S31. The fluorescence quantum yields of LP/Eu/DPA in aqueous solution.**

**Figure S32. The prompt photoluminescence spectra of (a) DPA added into G aqueous solution. (b) The luminescent quenching efficiency for the emission of the G/LP with the DPA at 380 nm and 516 nm.**

**Figure S33. The luminescent quenching efficiency for the emission of the G/LP/Eu with the DPA at 380 nm and 516 nm.**

**Figure S34.** **(a) The energy gap between S_1_ and T_1_ in the presence of different background charges; (b) Schematic diagram of the change in the energy gap between S_1_ and T_1_ with the magnitude of background charge; (c) Locations of background charge addition, electrostatic potential diagrams of the molecule under different background charges, and the charge distribution of key atoms in the molecule.; (d) Molecular structures under different background charges and the corresponding Gibbs free energies (energies based on the structure at 0 background charge).**

**Experimental section**

All chemicals were commercially available unless noted otherwise. 4-(4-bromophenyl) pyridine was purchased from bidepharm and bromoethane was purchased from Heowns. NMR spectra were recorded on a Bruker AV500 spectrometer. The steady-state fluorescence data were collected from the Varian Cary Eclipse fluorescence spectrometer and HITACHI fluorescence spectrophotometer (F-4600S). UV–vis spectra and optical transmittance were recorded in a quartz cell (light path, 10 mm) on a Shimadzu UV-3600 spectrophotometer equipped with a PTC-348WI temperature controller. Photoluminescence spectra and fluorescence lifetimes were measured by means of time–correlated single–photon counting on a FLS1000 instrument (Edinburg Instruments, Livingstone, UK). High-resolution transmission electron microscopy images were acquired using a Tecnai 20 high–resolution transmission electron microscope operating at an accelerating voltage of 200 keV; The sample was prepared by dropping the solution onto a copper grid, which was then air–dried. The zeta potentials were determined on a NanoBrook 90Plus at 298 K. Ion exchange was tested using Shimadzu's X-ray photoelectron spectrometer. Electrospray ionization mass spectra were measured with an Agilent 6520 Q-TOF-MS instrument. Geometry optimization of the ground state (S_0_) of the G structure was performed using the M06-2X functional^[1a]^, the 6-31G(d)^[1b]^ basis set, and the PCM^[1c]^ solvent model with water as the solvent. Additionally, DFT-D3^[1d]^ dispersion correction was applied during the structural optimization. Based on the optimized ground state structure of the G molecule, single-point energy calculations were conducted for its first excited singlet state (S_1_) and first excited triplet state (T_1_) using Time-Dependent Density Functional Theory (TD-DFT)^[1e]^, adopting the M06-2X functional, the 6-31G(d) basis set, and the PCM solvent model with water as the solvent. The difference in single-point energies between the S_1_ and T_1_ states represents the energy level difference between S_1_ and T_1_. All calculations were performed using the Gaussian 16 program^[1f]^, and the electrostatic potential maps of the guest molecule G under various background charge conditions were visualized using Multiwfn^[1g-1h]^ and GaussView 6.

Figure S1 The Synthetic route of G.

**Preparation of G_1_**.

3-Bromopropan-1-amine hydrobromide (0.23 g, 1.00 mmol) was added to a solution of 4-(4-bromophenyl) pyridine (0.47 g, 2.00 mmol) in acetonitrile (50 mL). The solution was heated under reflux for 2 h, during which time a large amount of precipitate formed. The reaction mixture was allowed to cool to room temperature and then filtered, and the obtained solid was washed thoroughly with acetonitrile to afford G as a pale yellow solid (0.15 g, 67%). ^1^H NMR (500 MHz, D_2_O, 25 °C) δ 8.75 (d, J = 7.0 Hz, 2H), 8.35 (d, J = 6.9 Hz, 2H), 7.71–7.70 (m, 4H), 4.60–4.57 (m, 2H), 3.04–3.01 (m, 2H), 2.34–2.28 (m, 2H). ^13^C NMR (500 MHz, D_2_O, 25 °C) δ 156.09, 144.24, 132.78, 132.75, 129.61, 126.71, 125.23, 57.68, 36.20, 28.32. HRMS (ESI) for C_14_H_16_Br_2_N_2_: calcd. [M–HBr–Br]^+^: 291.0497, found: 291.0497.

**Figures**

**
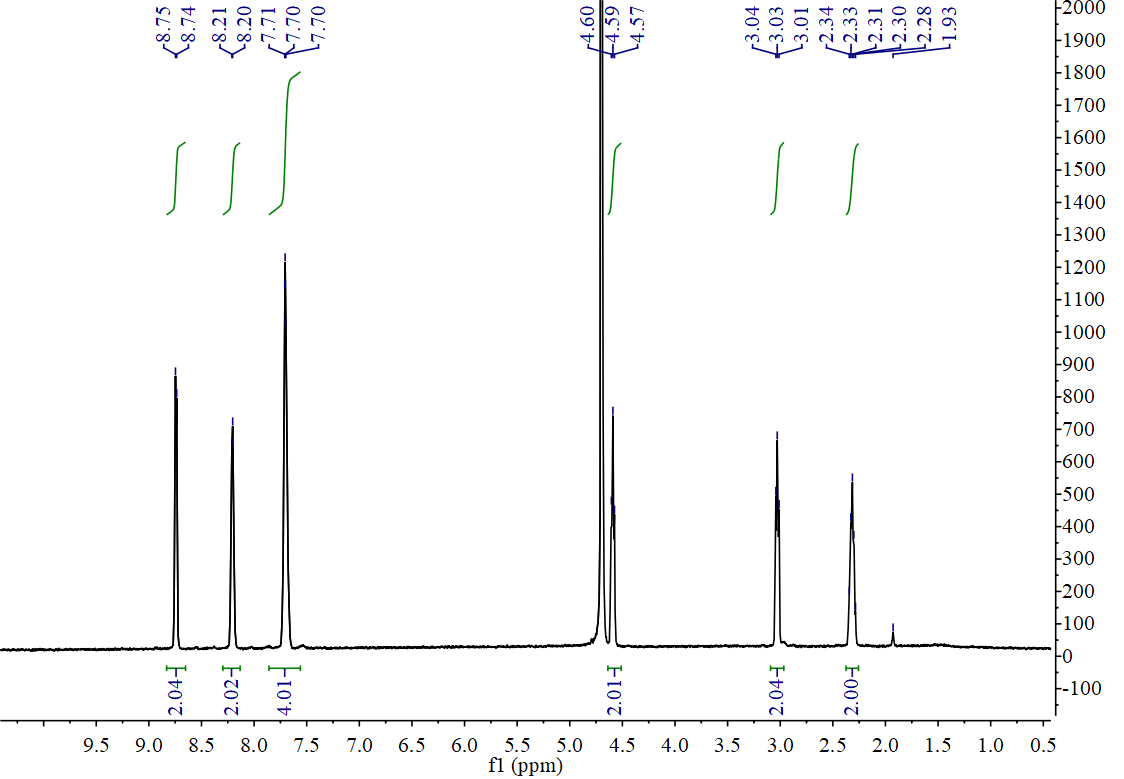
**

**Figure S2.** ^1^H NMR (500 MHz, D_2_O, 298 K) spectrum of compound G.

**
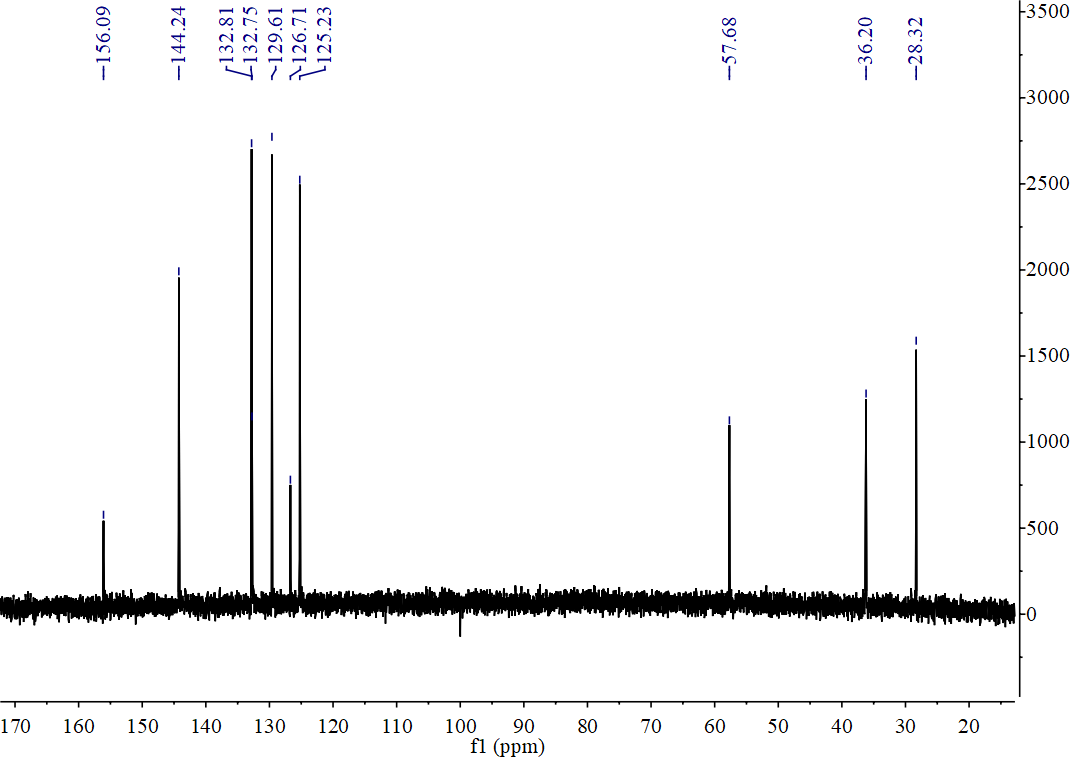
**

**Figure S3.** ^13^C NMR (125 MHz, D_2_O, 298 K) spectrum of compound G.


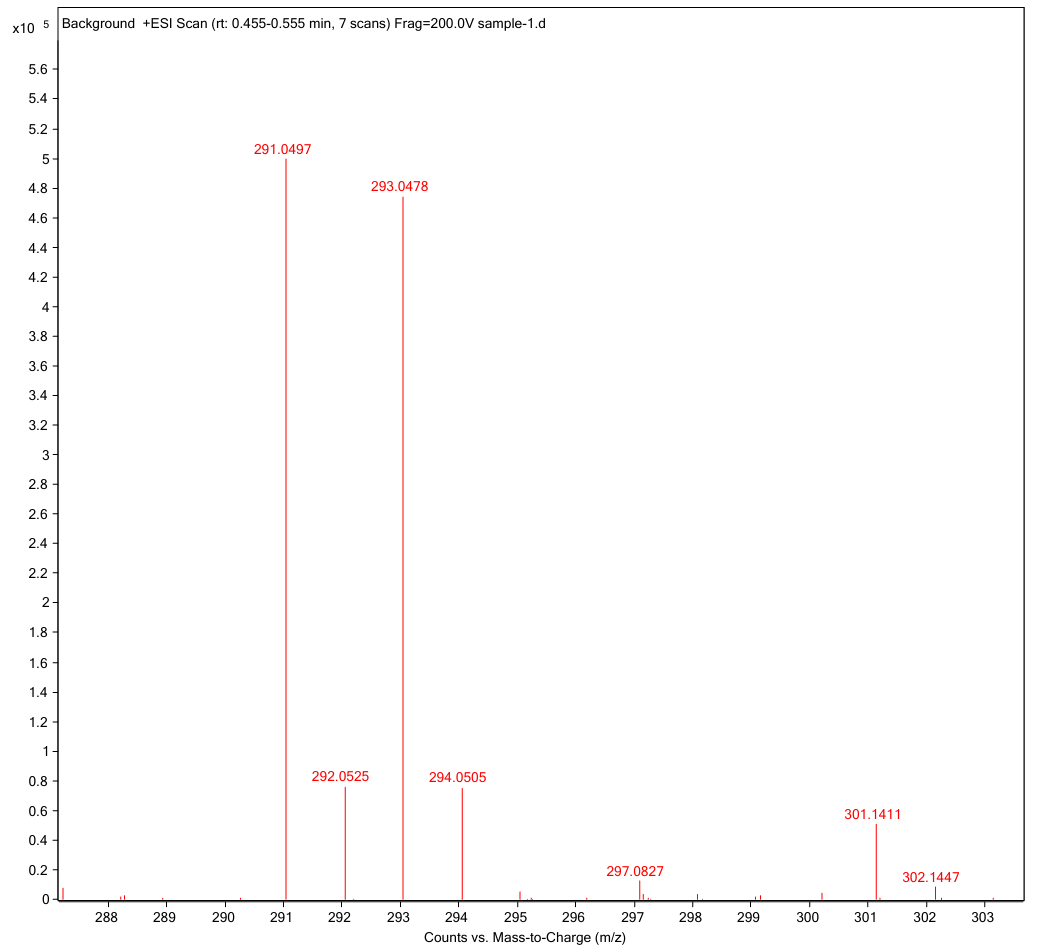


**Figure S4**. ESI mass spectrum of G.

**Figure S5.** The phosphorescence lifetime decay curves of G/LP/N_2_ in aqueous solution ([G] = 0.01 mM, LP = 2 wt%, λ_ex_ = 300 nm, λ_em_ = 516 nm).

**
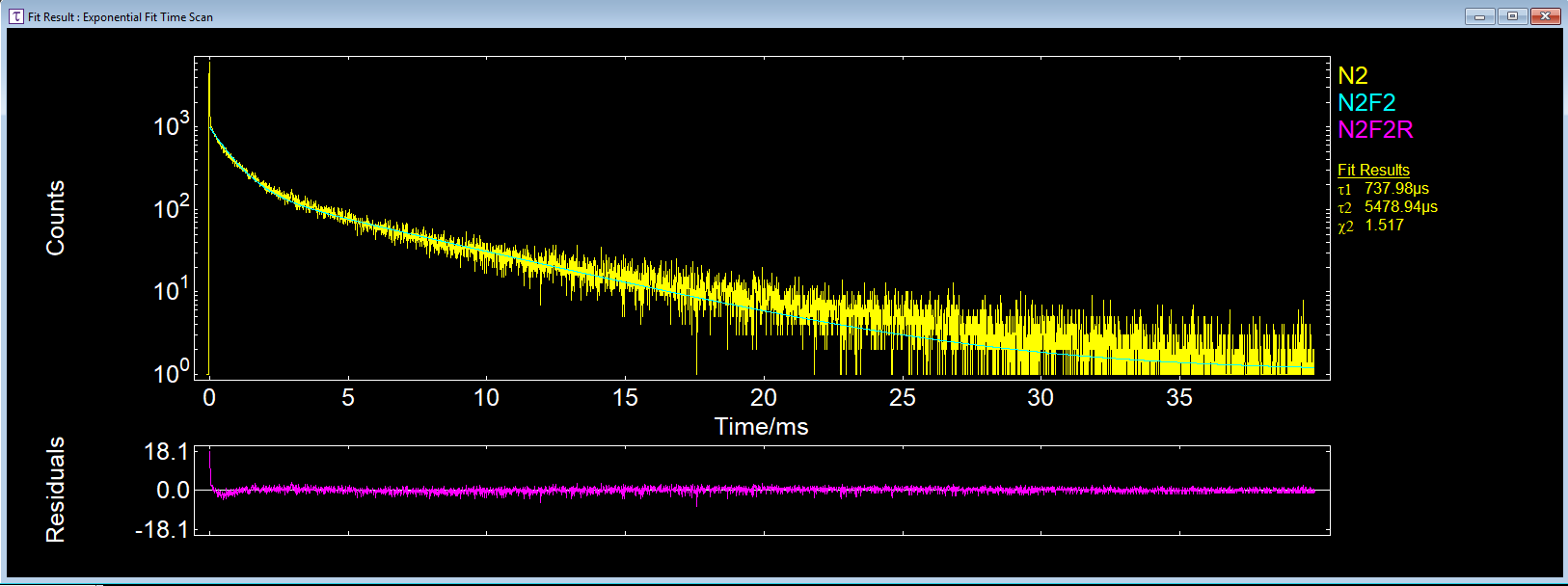
**

**Figure S6.** The phosphorescence lifetime decay fitting curves of G/LP/N_2_ in aqueous solution ([G] = 0.01 mM, LP = 2 wt%, λ_ex_ = 300 nm, λ_em_ = 516 nm).

**
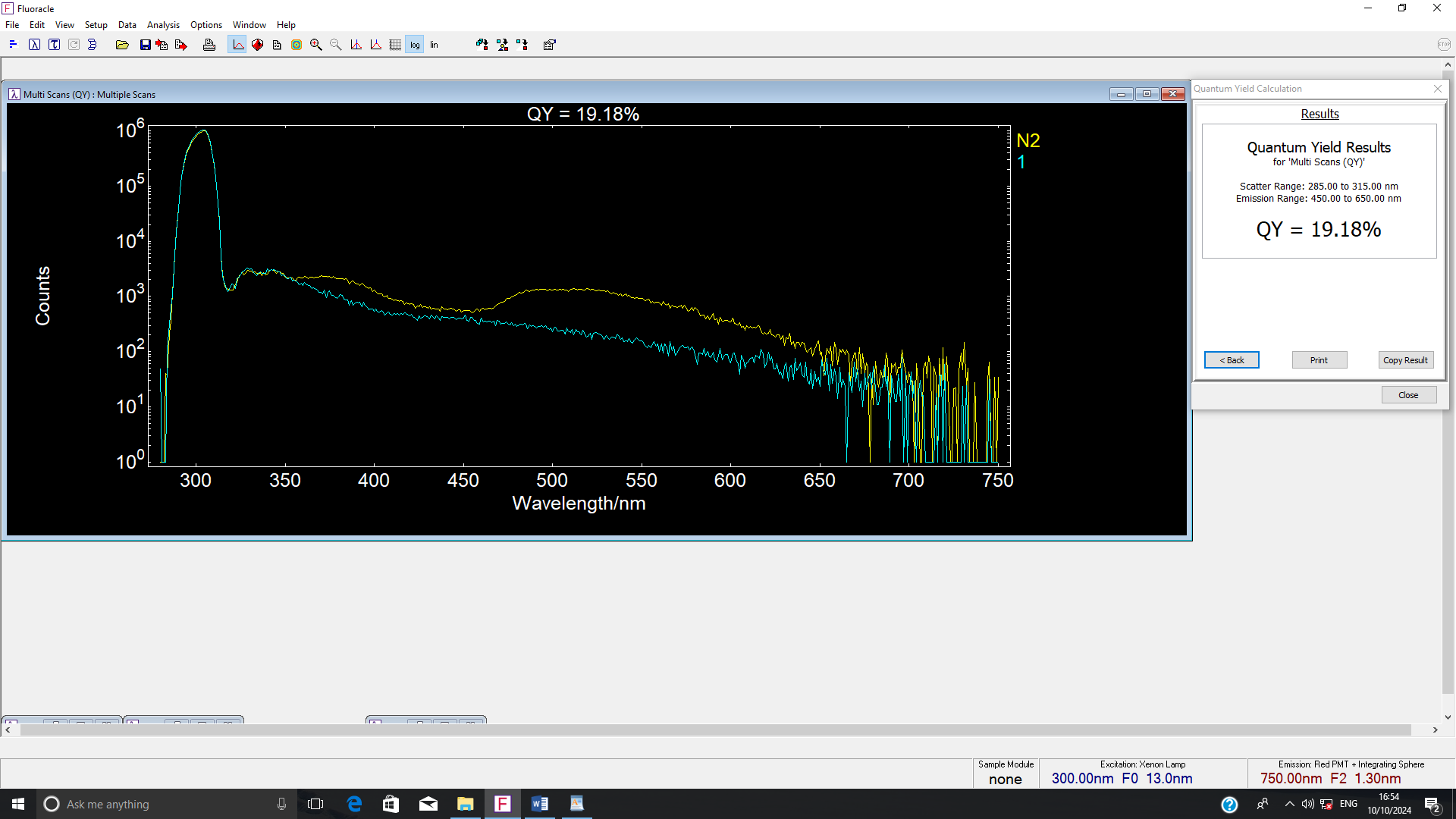
**

**Figure S7.** The phosphorescence quantum yields of G/LP/N_2_ in aqueous solution (The value ranges from 450 nm to 650 nm, [G] = 0.01 mM, LP = 2 wt%, λ_ex_ = 300 nm).

**Figure S8.** Phosphorescence emission spectra (delay 0.1s) of G in water before and after N_2_ bubbling at 298 K ([G] = 0.01 mM, λ_ex_ = 300 nm).


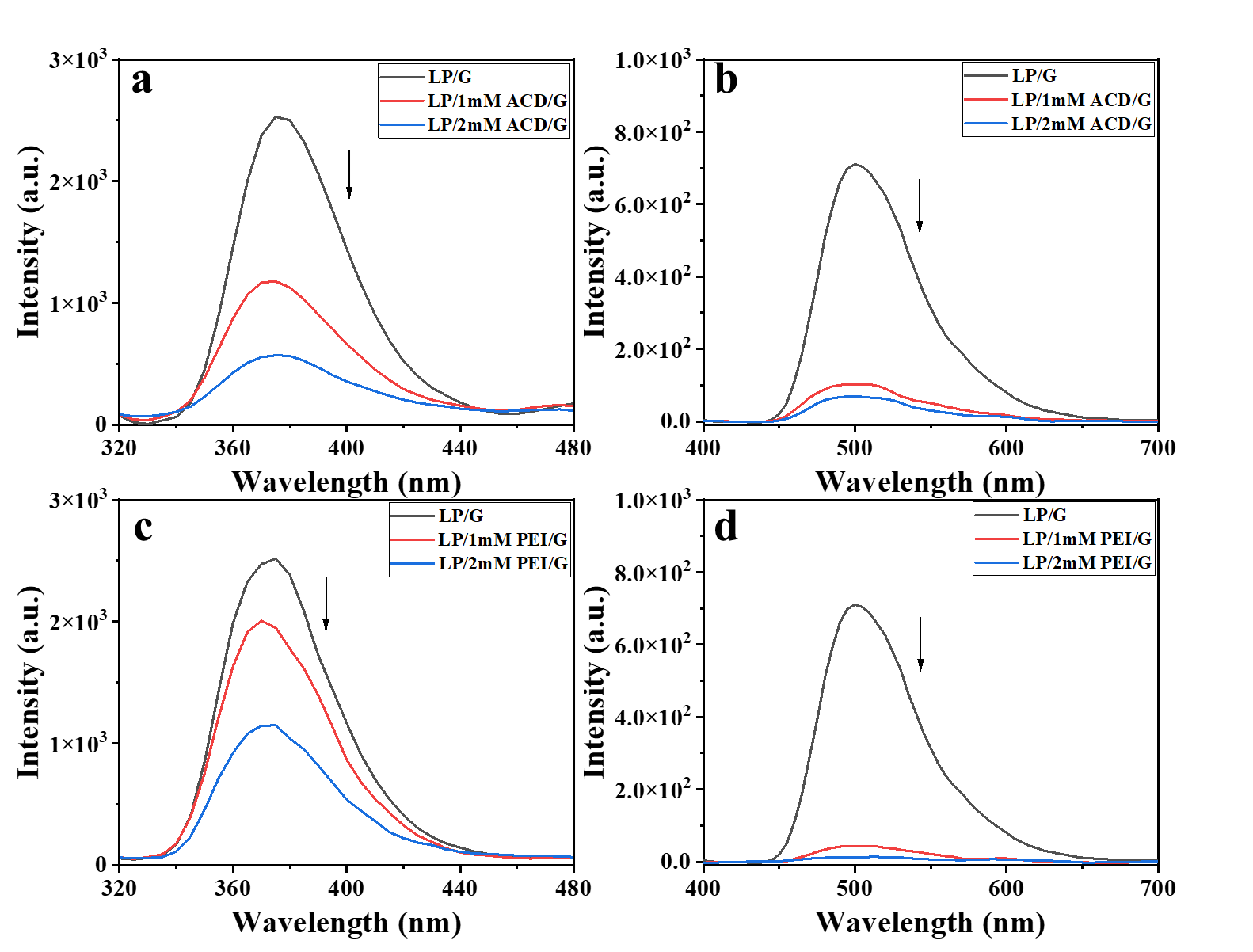


**Figure S9.** (a) Fluorescence spectra and (b) phosphorescence spectra of LP/ACD/G, (c) Fluorescence spectra and (d) phosphorescence spectra of LP/PEI/G. ([G] = 0.01 mM, λ_ex_ = 300 nm).


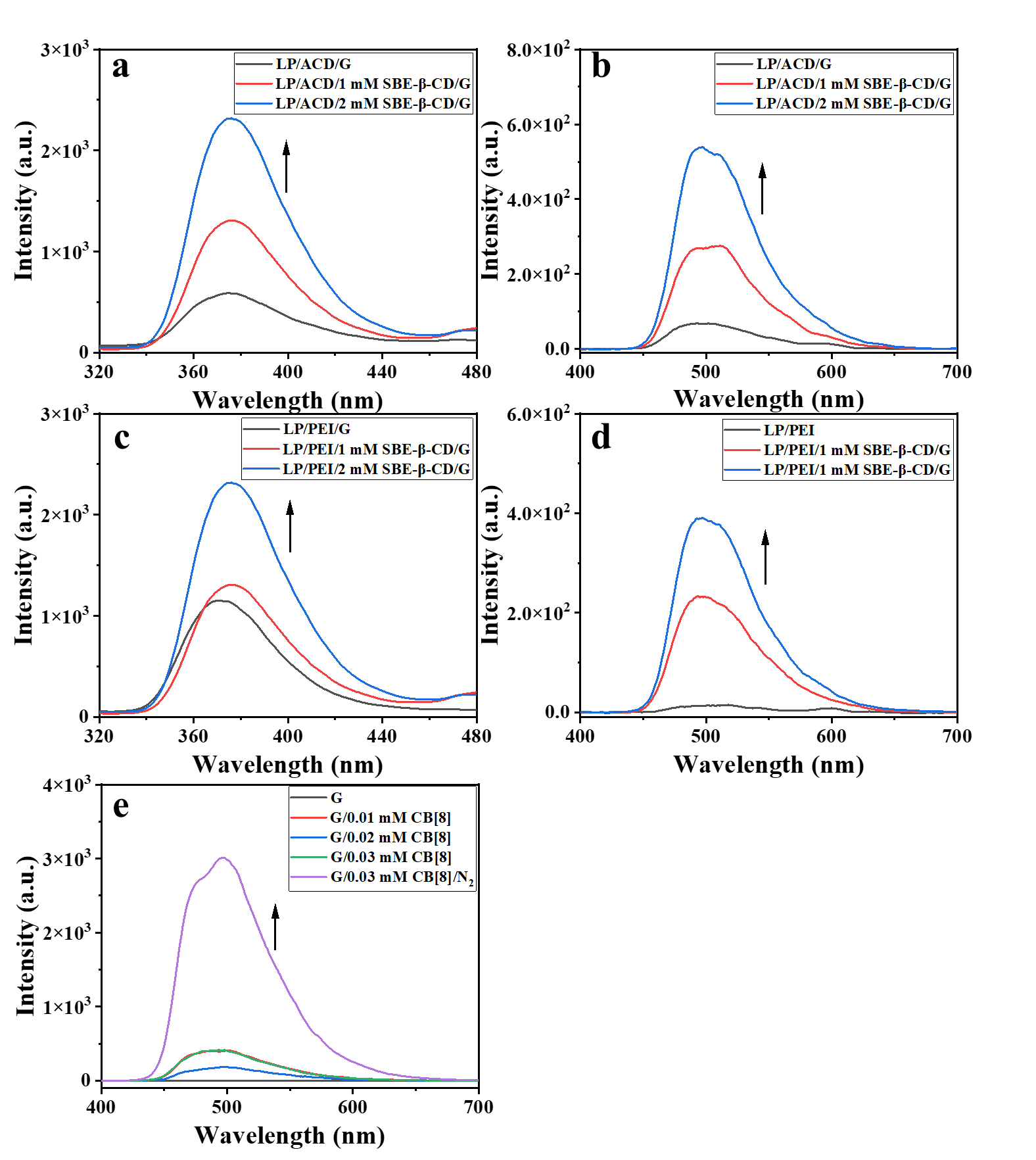


**Figure S10.** (a) Fluorescence spectra and (b) phosphorescence spectra of LP/ACD/SBE-*β*-CD/G, (c) Fluorescence spectra and (d) phosphorescence spectra of LP/PEI/ SBE-*β*-CD/G, (e) Phosphorescence emission spectra (delay 0.1s) of G/CB[8] in water before and after N_2_ bubbling at 298 K ([G] = 0.01 mM, λ_ex_ = 300 nm).


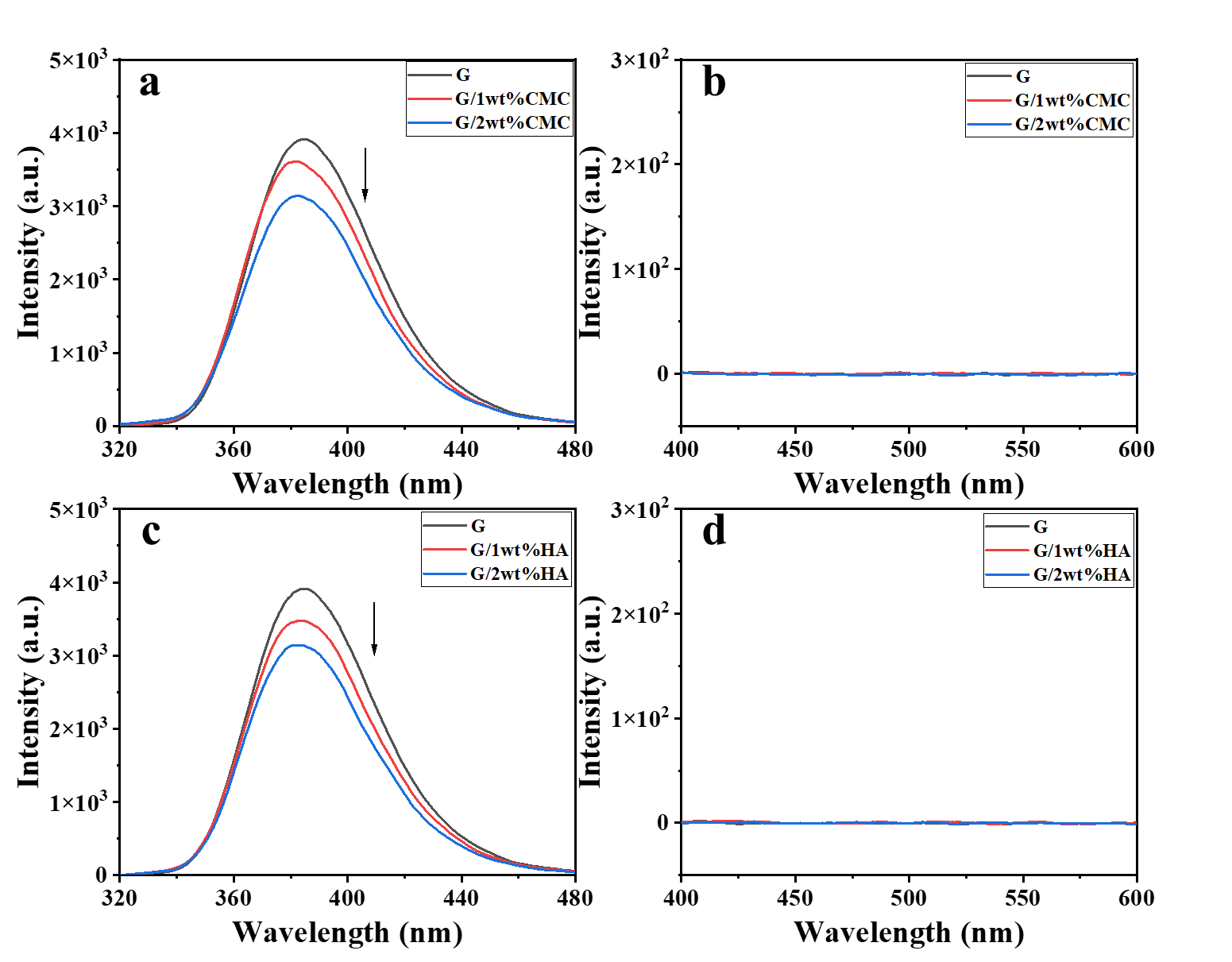


**Figure S11.** (a) Fluorescence spectra and (b) phosphorescence spectra of G/CMC, (c) Fluorescence spectra and (d) phosphorescence spectra of G/HA. ([G] = 0.01 mM, λ_ex_ = 300 nm).

**
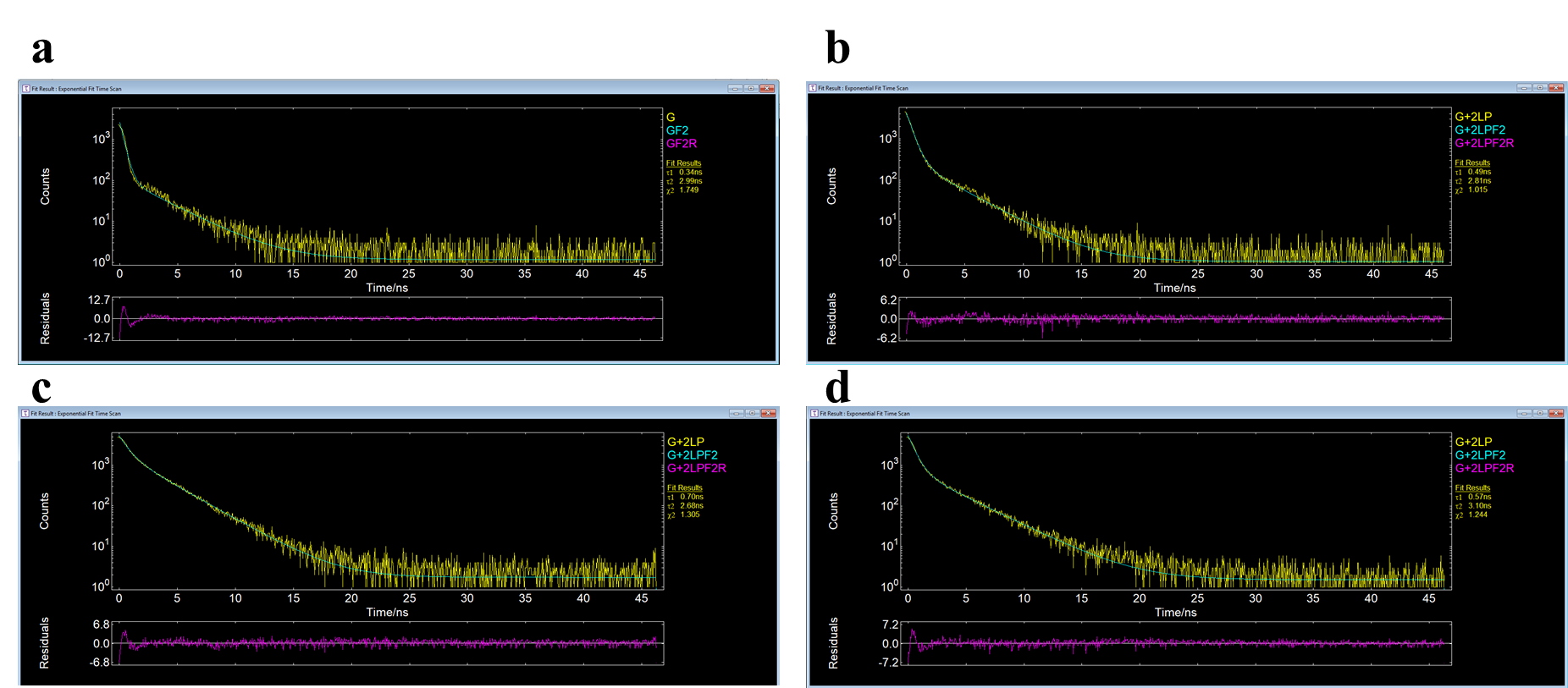
**

**Figure S12.** The fluorescence lifetime decay fitting curves of(a)G, (b) G/LP-0 min, (c) G/LP-24 min and (d) G/LP-60 min in aqueous solution ([G] = 0.01 mM, LP = 2 wt% λ_ex_ = 300 nm, λ_em_ = 380 nm).

**
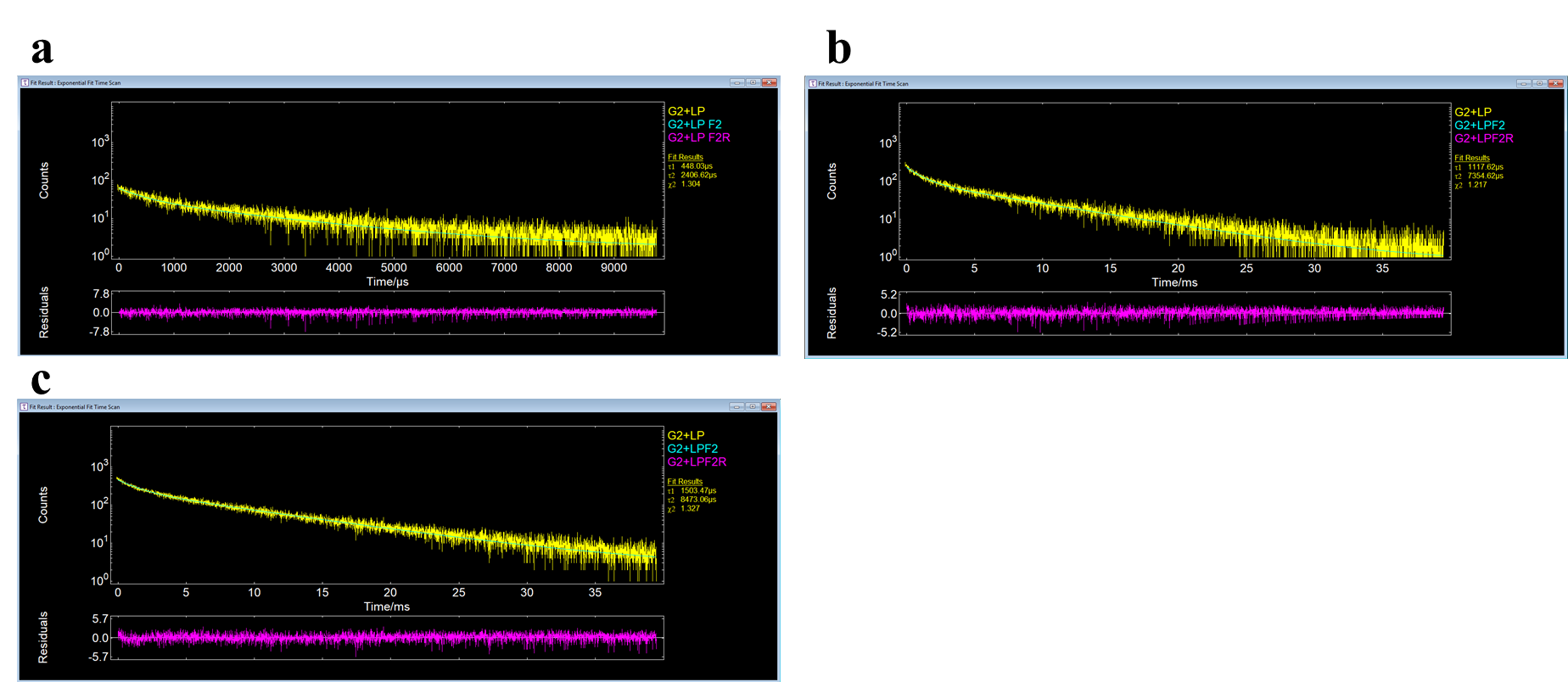
**

**Figure S13.** The phosphorescence lifetime decay fitting curves of (a) G/LP-0 min, (b) G/LP-60 min and (c) G/LP-60 min in aqueous solution ([G] = 0.01 mM, LP = 2 wt%, λ_ex_ = 300 nm, λ_em_ = 516 nm).

**
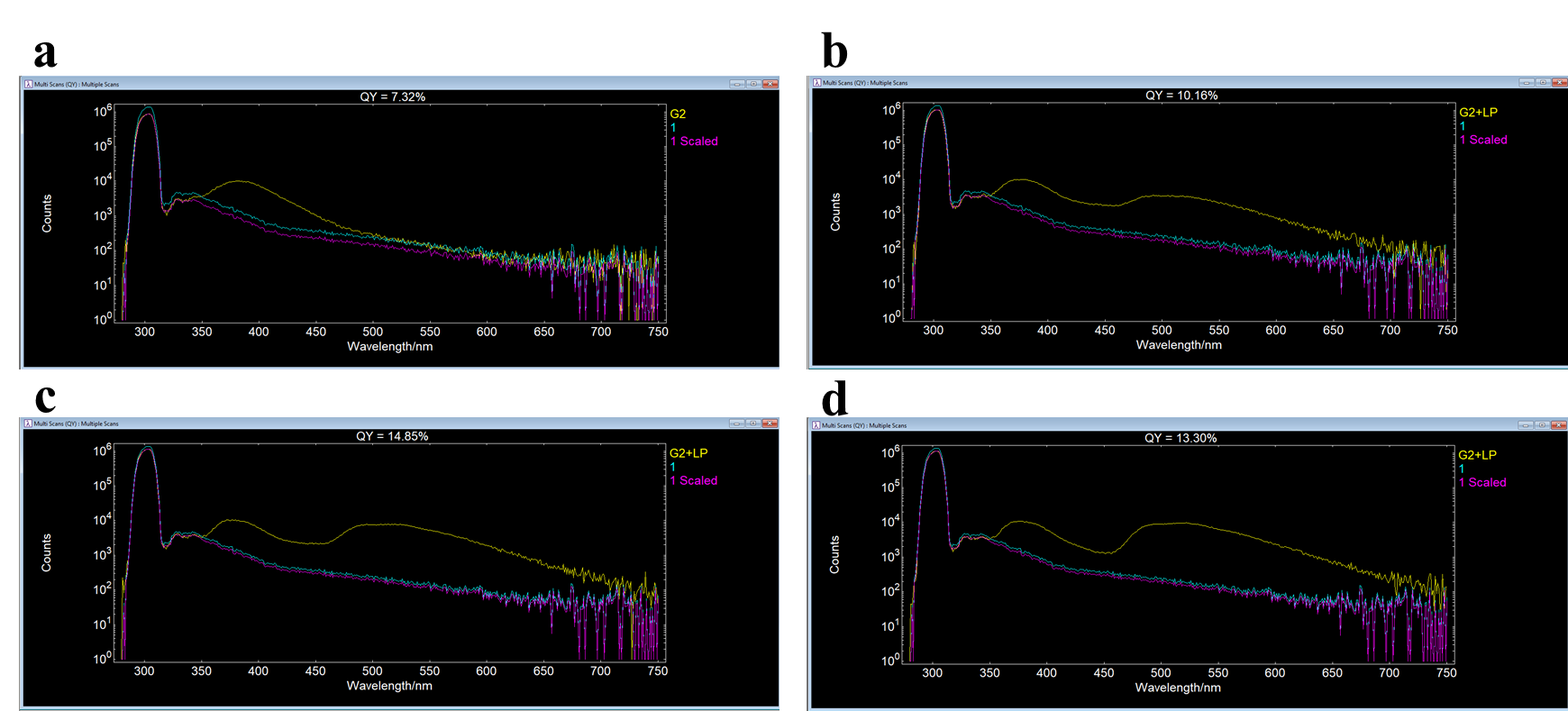
**

**Figure S14.** The fluorescence quantum yields of (a) G, (b) G/LP-0 min, (c) G/LP-24 min and (d) G/LP-60 min in aqueous solution (The value ranges from 350 nm to 450 nm, [G] = 0.01 mM, LP = 2 wt%, λ_ex_ = 300 nm).

**
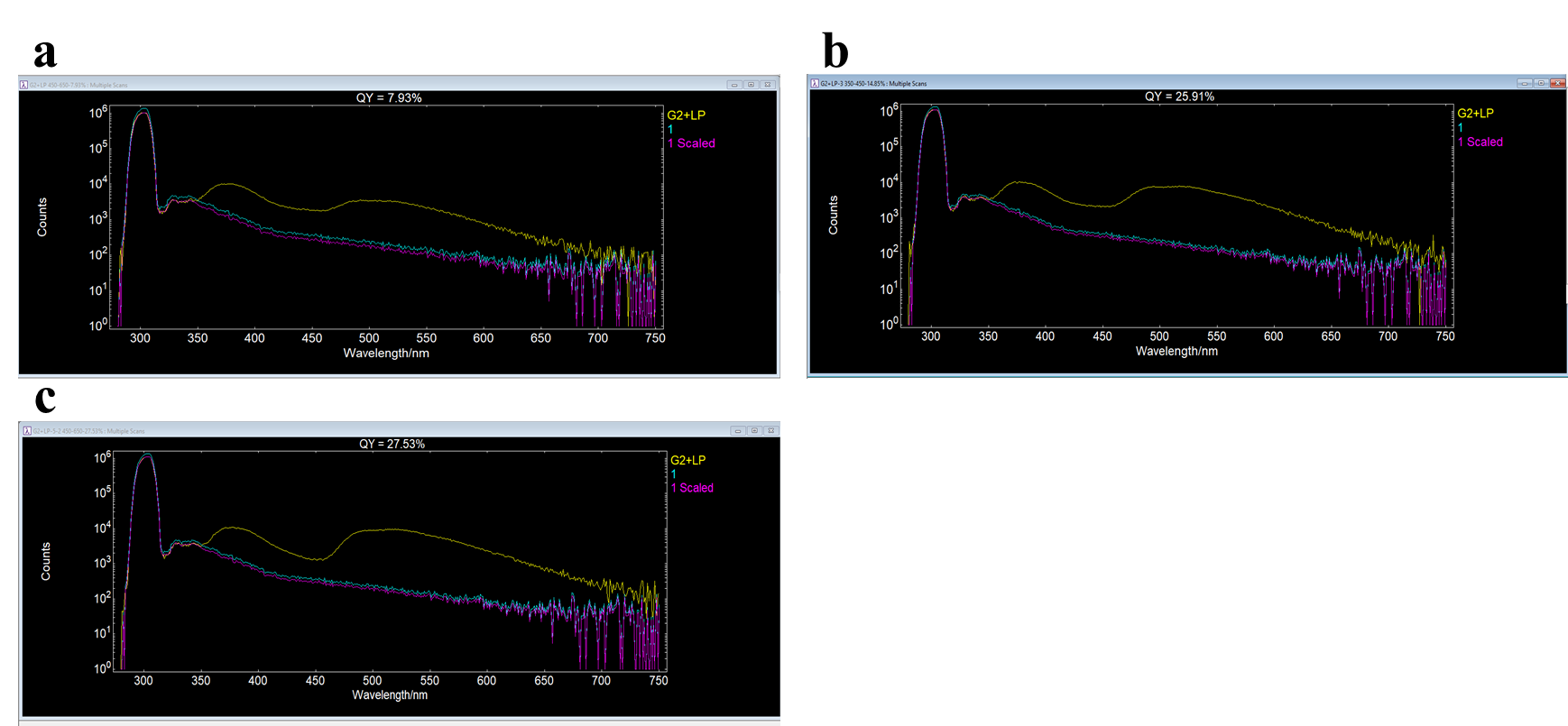
**

**Figure S15**. The phosphorescence quantum yields of (a) G/LP-0 min, (b) G/LP-24 min and (c) G/LP-60 min in aqueous solution (The value ranges from 450 nm to 650 nm, [G] = 0.01 mM, LP = 2 wt%, λ_ex_ = 300 nm).


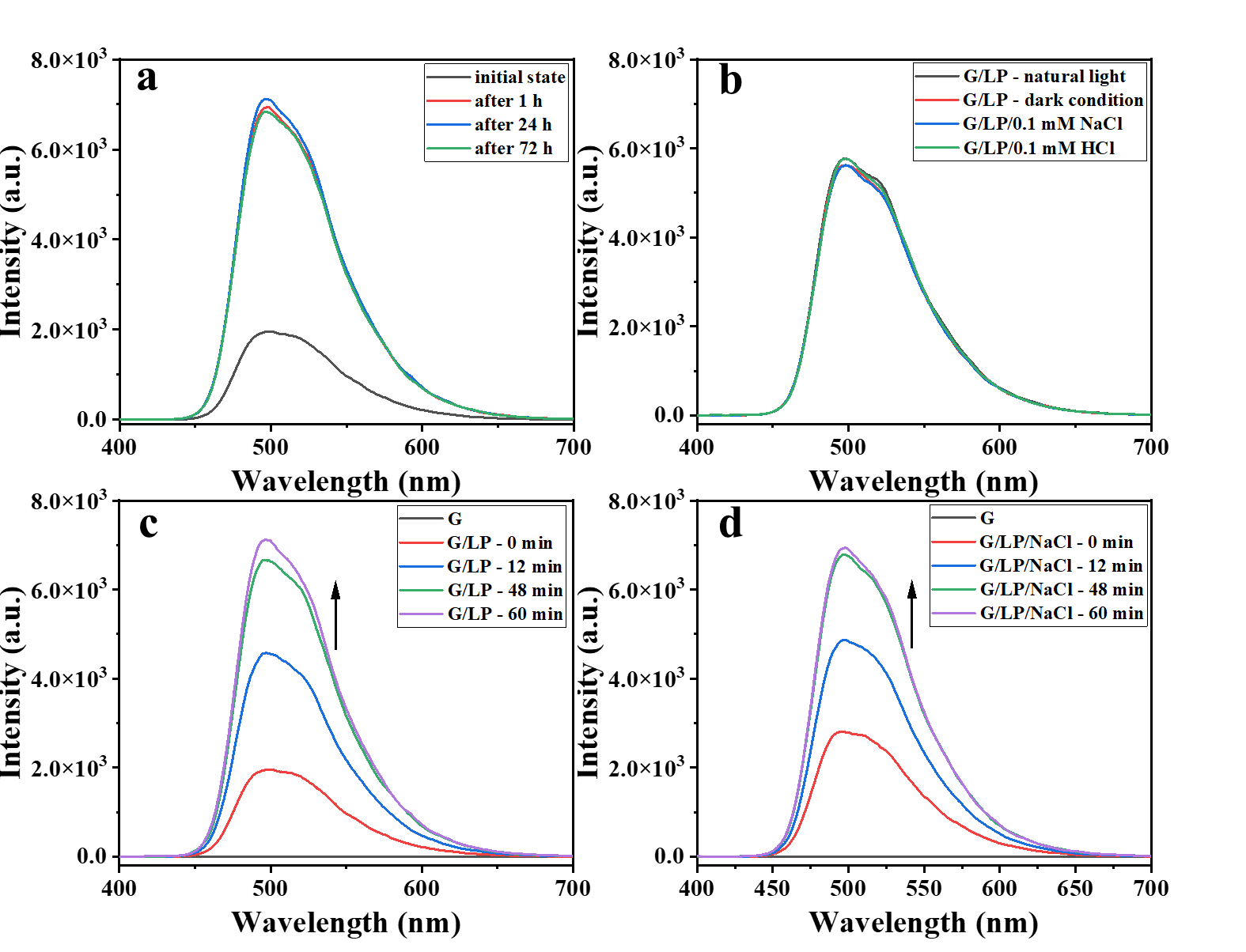


**Figure S16**. Phosphorescence emission spectra (delay 0.1s) of (a) G/LP assembly solution under natural light for 72 hours at 298 K, (b) The phosphorescence emission spectra (delay 0.1s) of the G/LP assembly solution at 298 K under different conditions, (c) The dynamic Phosphorescence emission spectra (delay 0.1s) of G with LP in water at 298 K, (d) The dynamic Phosphorescence emission spectra (delay 0.1s) of G/LP/NaCl in water at 298 K ([G] = 0.01 mM, λ_ex_ = 300 nm).

**
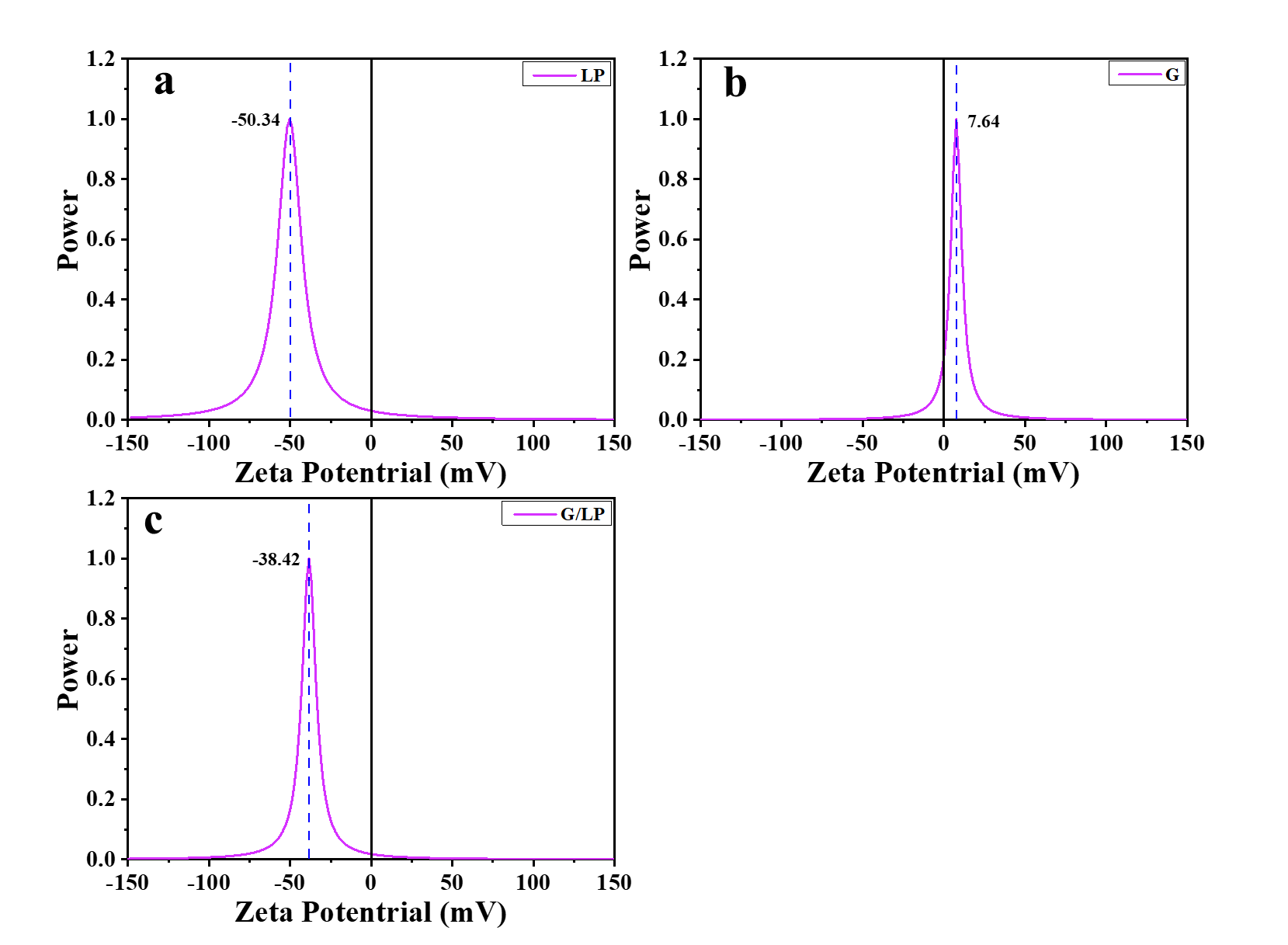
**

**Figure S17.** Zeta potential of (a) LP, (b) G and (c) G/LP in aqueous solution at 298 K ([G] = 0.01 mM, LP = 2 wt%).

**
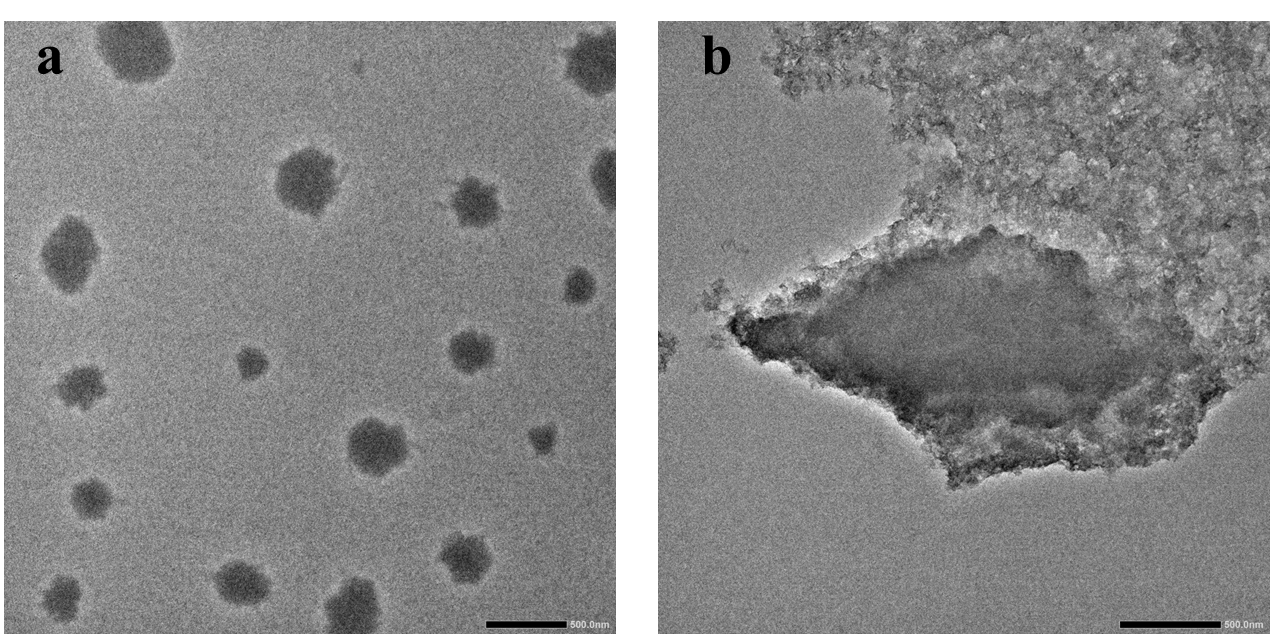
**

**Figure S18.** Transmission electron microscopy (TEM) images of (a) G and (b) G/LP.

**Figure S19.** The fluorescence lifetime decay curves of LP/Eu in aqueous solution (λ_ex_ = 254 nm, λ_em_ = 616 nm).

**
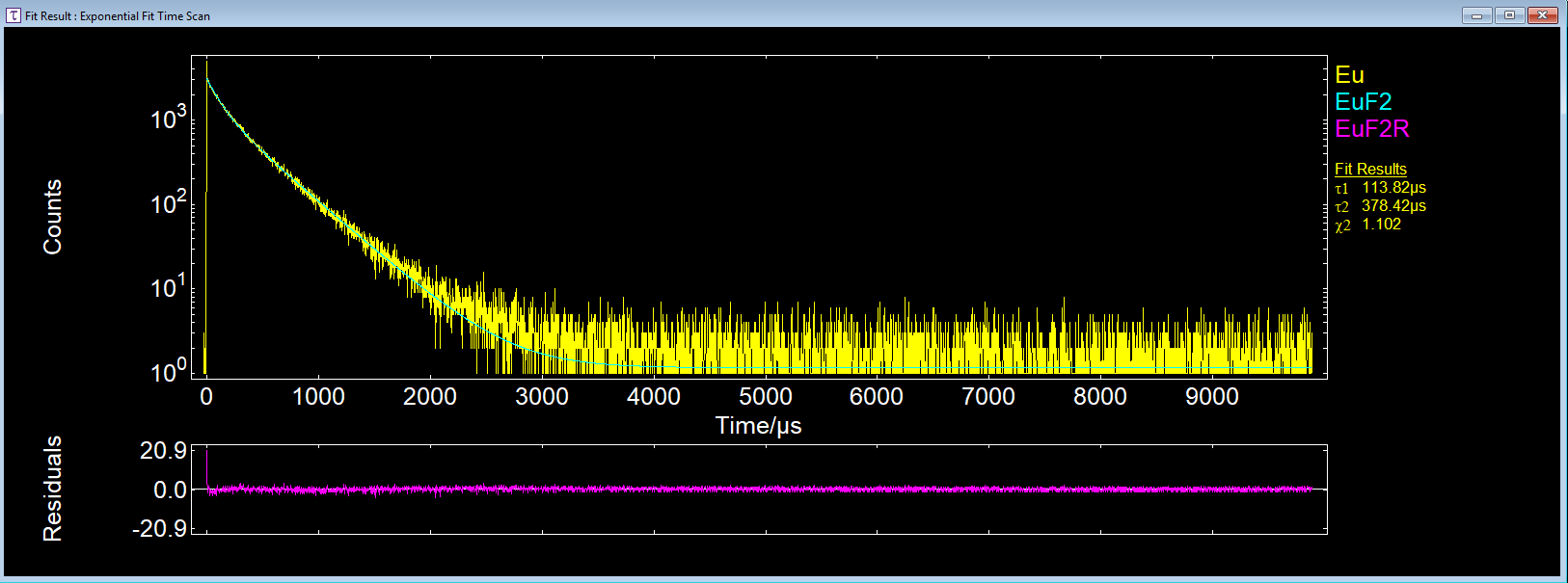
**

**Figure S20.** The fluorescence lifetime decay fitting curves of LP/Eu in aqueous solution (λ_ex_ = 254 nm, λ_em_ = 616 nm).

**
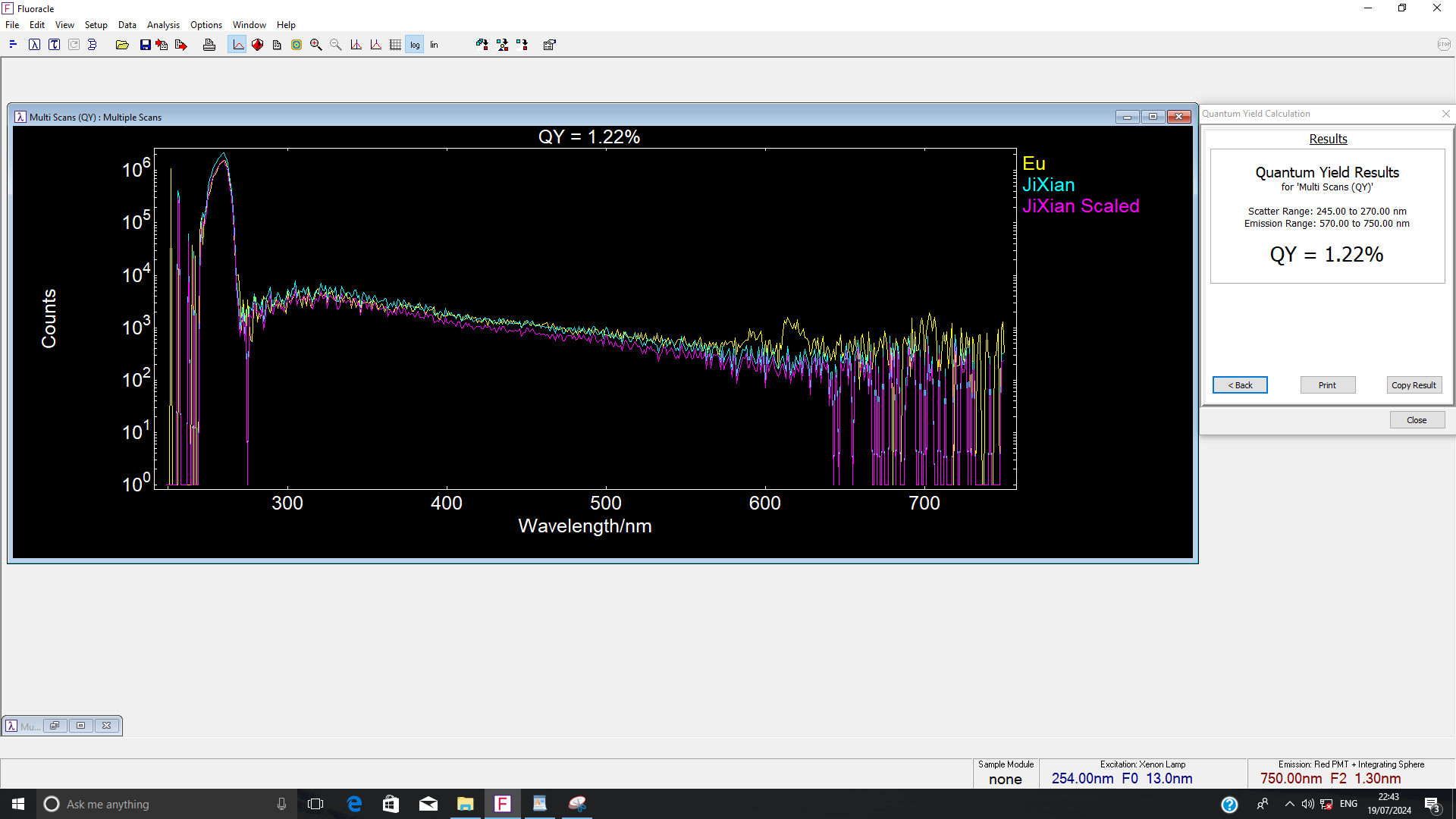
**

**Figure S21.** The fluorescence quantum yields of LP/Eu in aqueous solution (The value ranges from 570 nm to 750 nm, λ_ex_ = 300 nm).


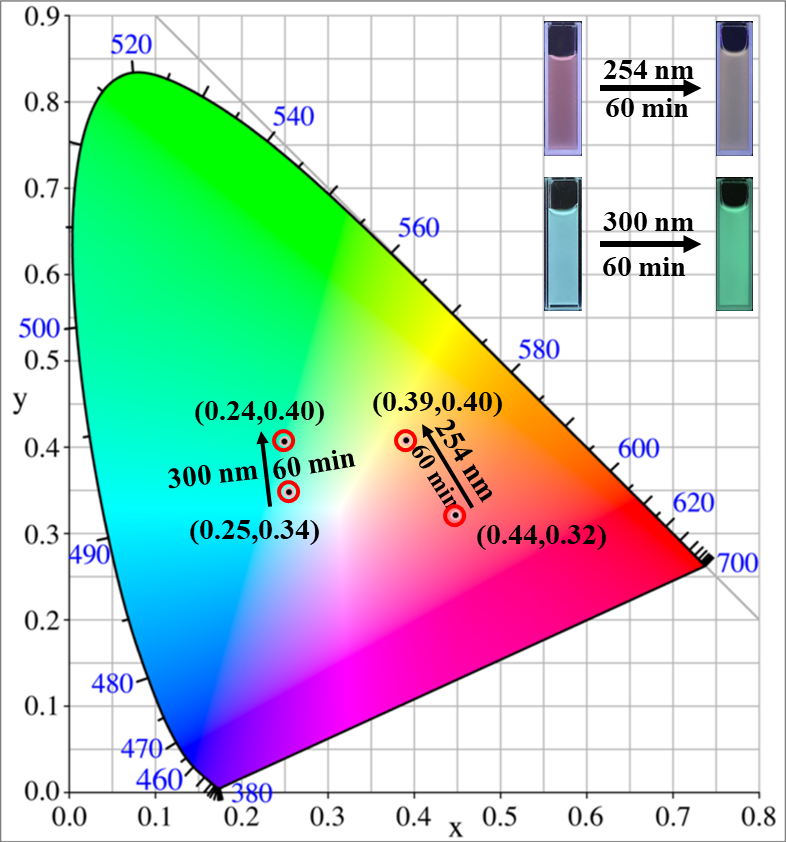


**Figure S22.** The corresponding CIE chromaticity graph of the dynamic changed spectra of G with the excess LP/Eu in water at 298 K (λ_ex_ = 300 nm).


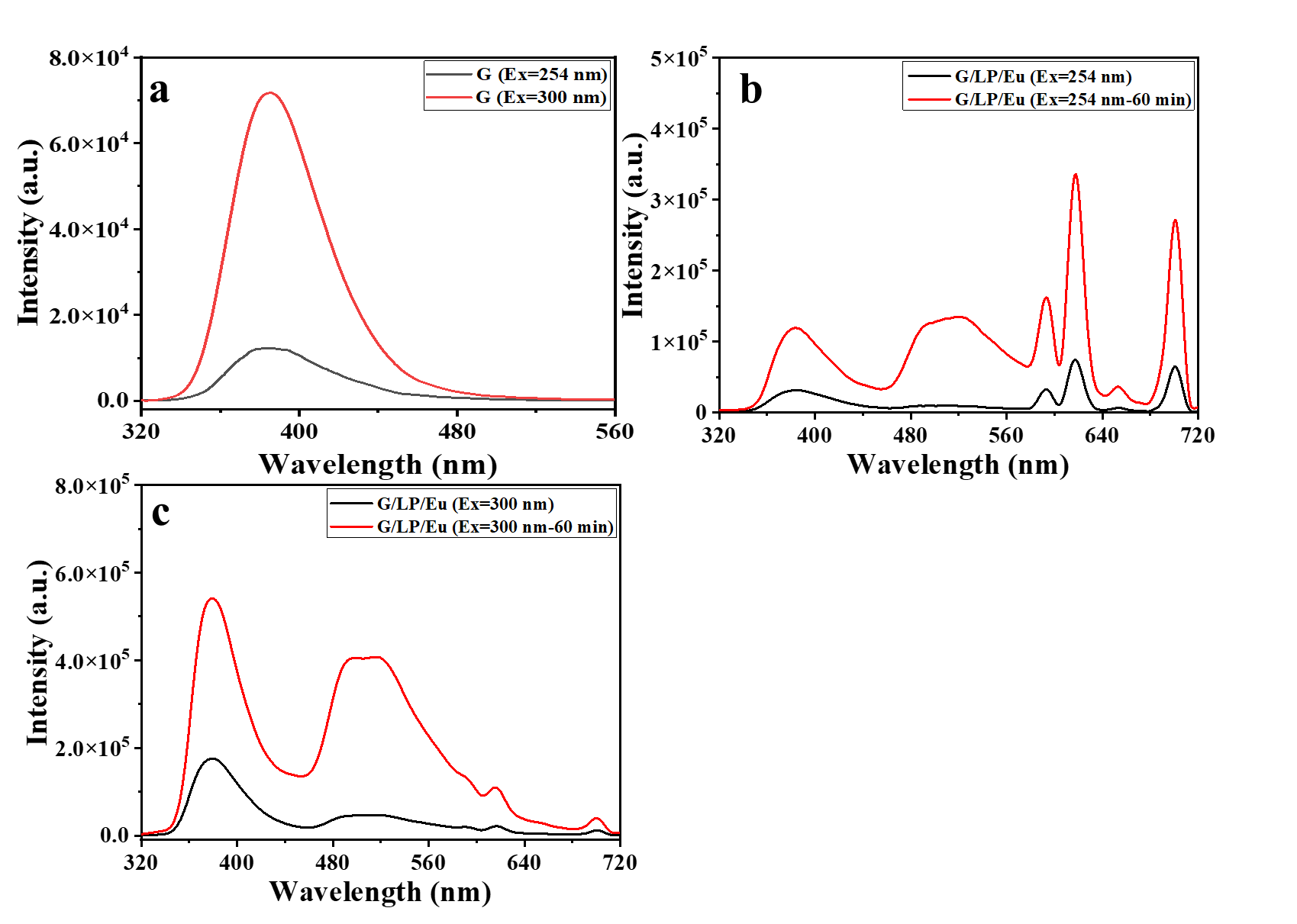


**Figure S23.** (a) The prompt photoluminescence spectra of G aqueous solution; (b) The dynamic prompt photoluminescence spectra of G aqueous solution with excess LP/Eu under 254 nm excitation; (c) The dynamic prompt photoluminescence spectra of G aqueous solution with excess LP/Eu under 300 nm excitation ([G] = 0.01 mM, LP/Eu = 0.5 wt%).

**
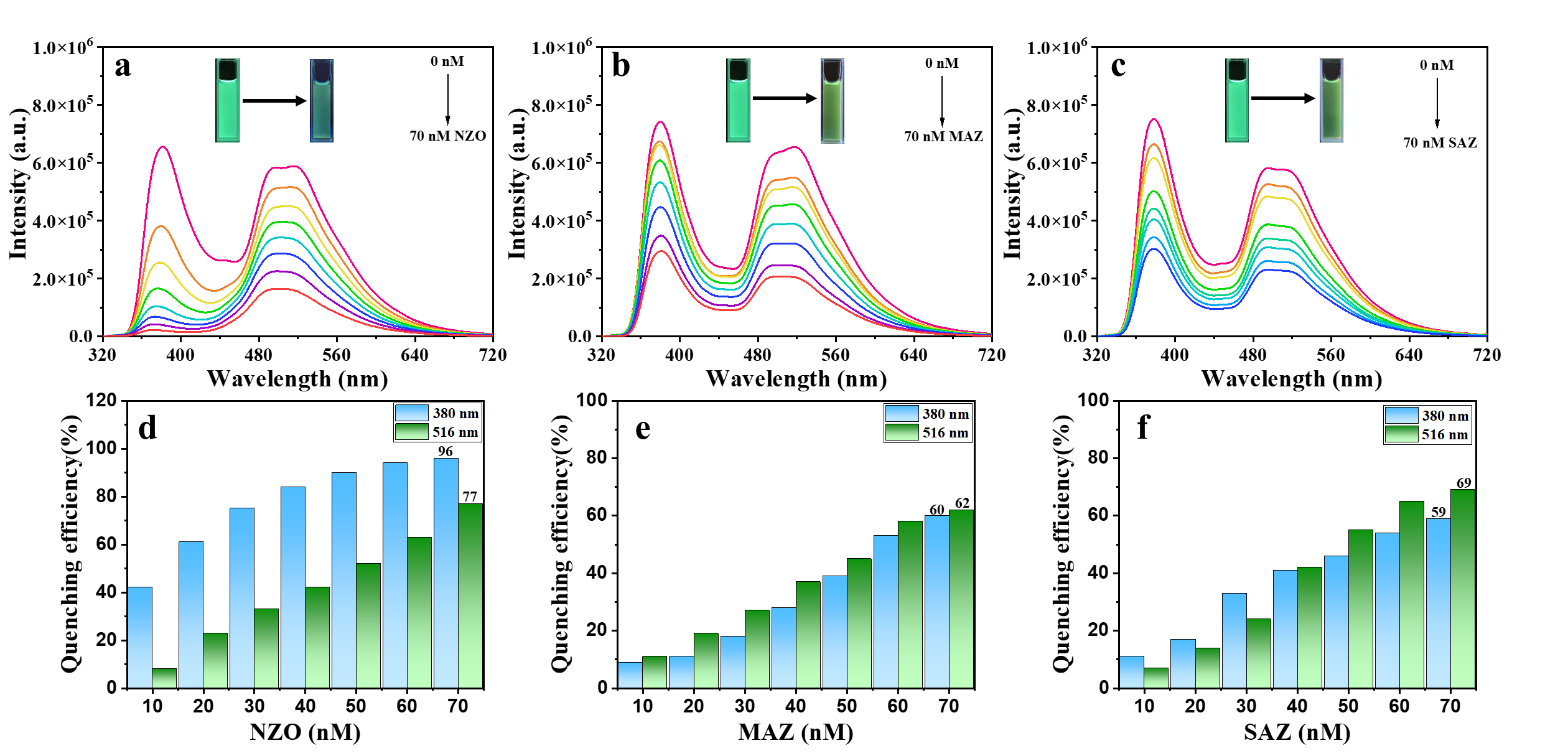
**

**Figure S24.** The prompt photoluminescence spectra of (a) NZO, (b) MAZ, and (c) SAZ added to G aqueous solution; The luminescence quenching efficiencies of (d) NZO, (e) MAZ, and (f) SAZ corresponding to G/LP (λ_em_ = 380 nm and λ_em_ = 516 nm) ([G] = 0.01 mM, LP = 2 wt%, [NZO] = 70 nM, [MAZ] =70 nM, [SAZ] = 70 nM).

**
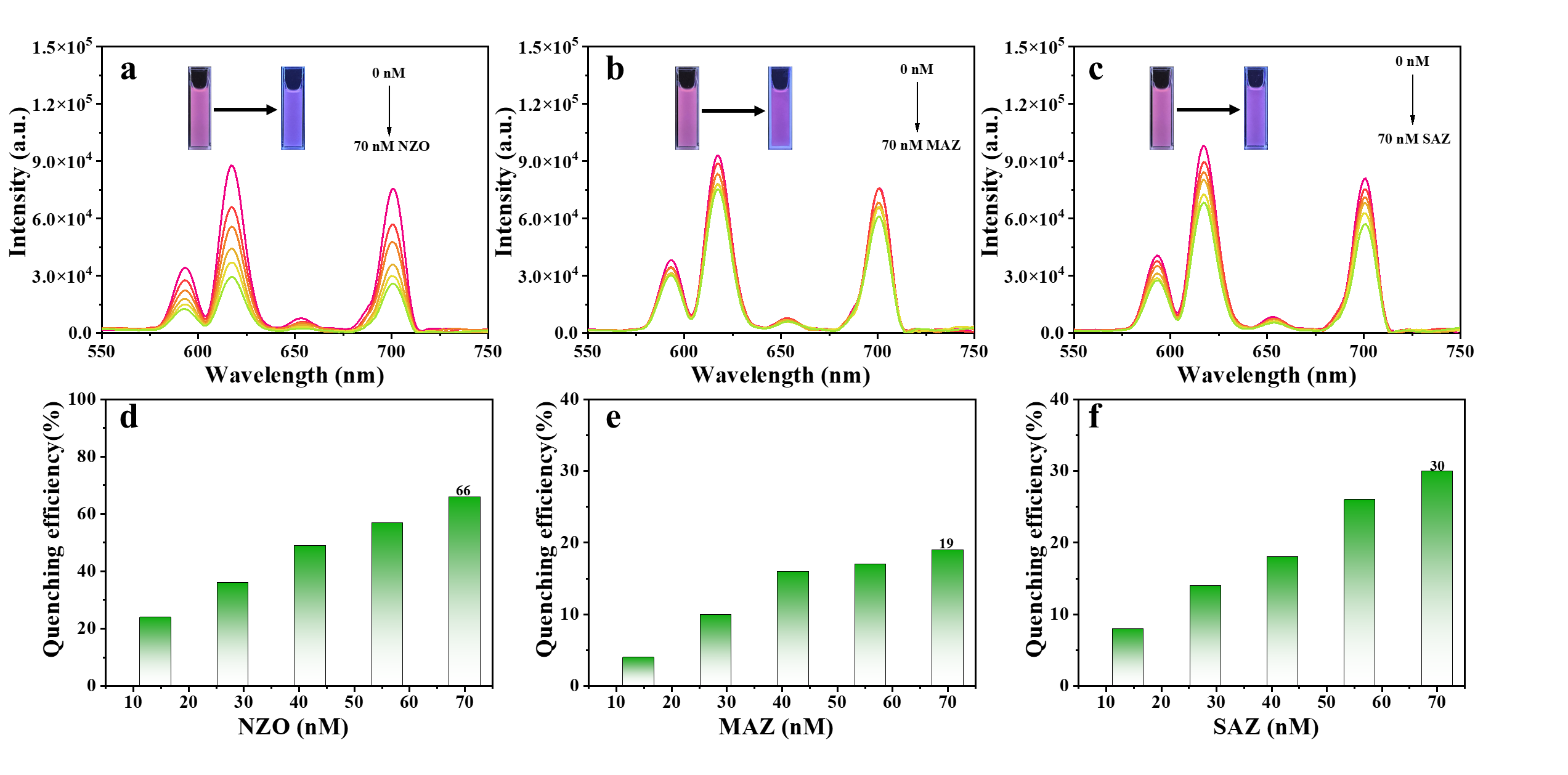
**

**Figure S25.** The prompt photoluminescence spectra of (a) NZO, (b) MAZ, and (c) SAZ added to LP/Eu aqueous solution; The luminescence quenching efficiencies of (d) NZO, (e) MAZ, and (f) SAZ corresponding to LP/Eu (λ_em_ = 616nm) (LP/Eu = 0.5 wt%, [NZO] = 70 nM, [MAZ] = 70 nM, [SAZ] = 70 nM).

**Figure S26.** The luminescence quenching efficiencies of NZO corresponding to G/LP (λ_em_ = 380 nm, λ_em_ = 516 nm and λ_em_ = 616 nm, LP/Eu = 0.2 wt%, [NZO] = 70 nM).

**
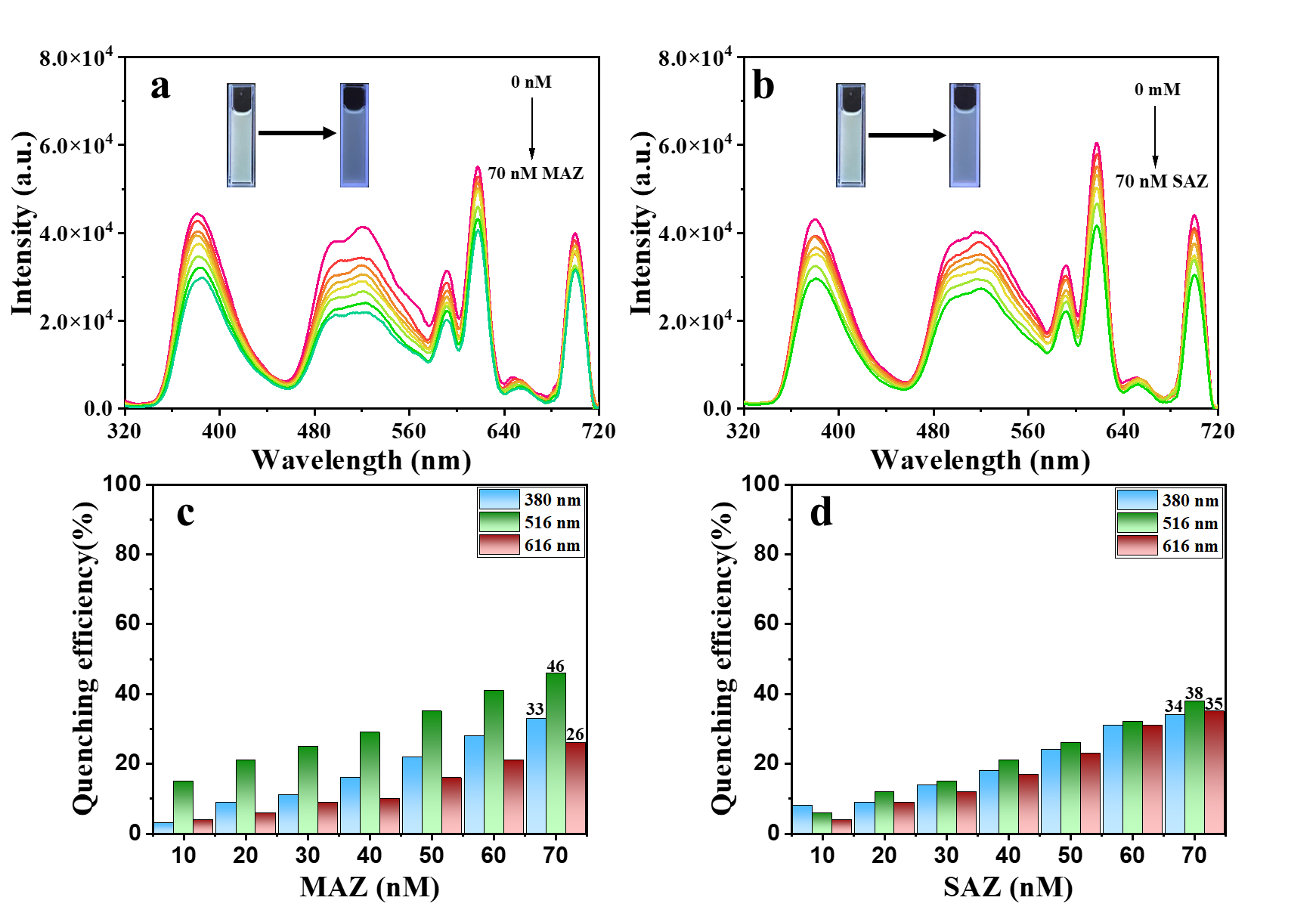
**

**Figure S27.** The prompt photoluminescence spectra of (a) MAZ, and (b) SAZ added to G/LP/Eu aqueous solution; The luminescence quenching efficiencies of (c) MAZ and (d) SAZ corresponding to G/LP/Eu (λ_em_ = 380 nm, λ_em_ = 516 nm and λ_em_ = 616 nm) ([G] = 0.01 mM, LP/Eu = 0.2 wt%, [MAZ] = 70 nM and [SAZ] = 70 nM).

**
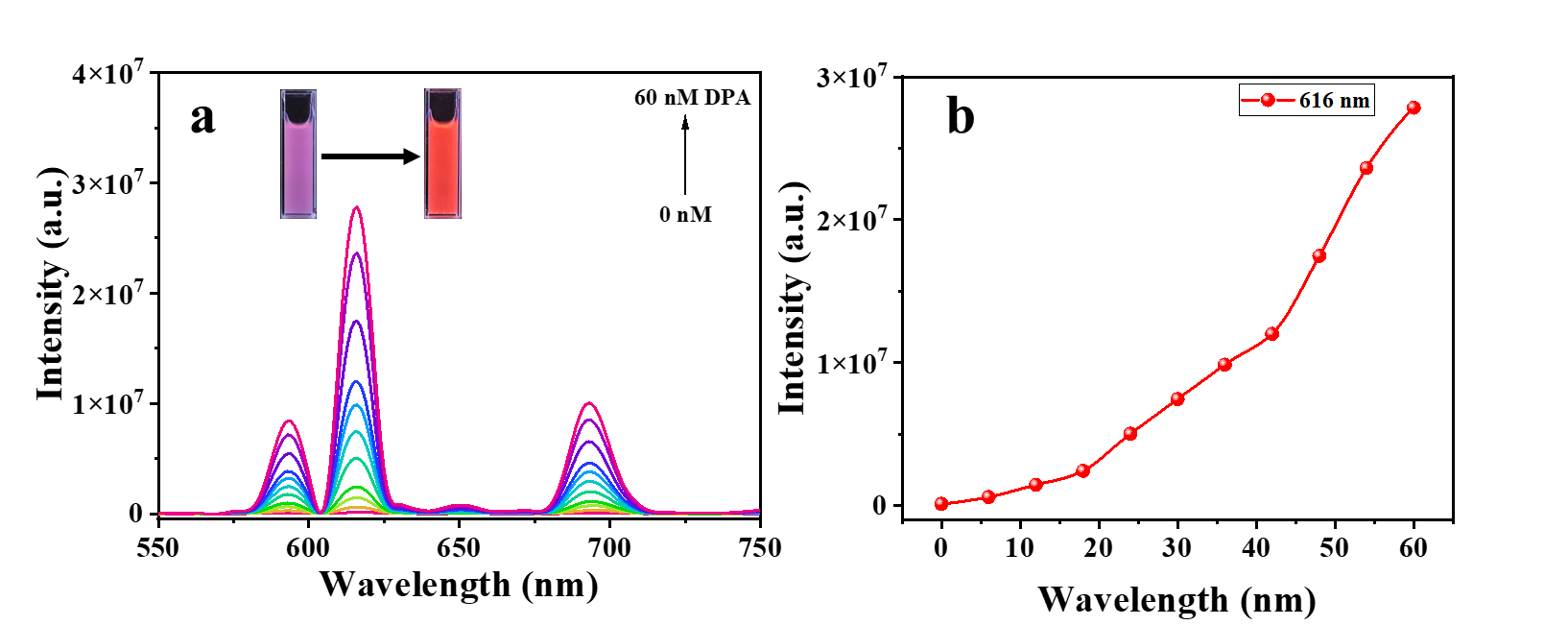
**

**Figure S28.** (a) The prompt photoluminescence spectra of (a) DPA added to LP/Eu aqueous solution. (b) DPA enhances the emission intensity curve of LP/Eu (LP/Eu = 0.5 wt%, [DPA] = 70 nM).

**Figure S29.** The fluorescence lifetime decay curves of LP/Eu/DPA in aqueous solution (λ_ex_ = 254 nm, λ_em_ = 616 nm).

**
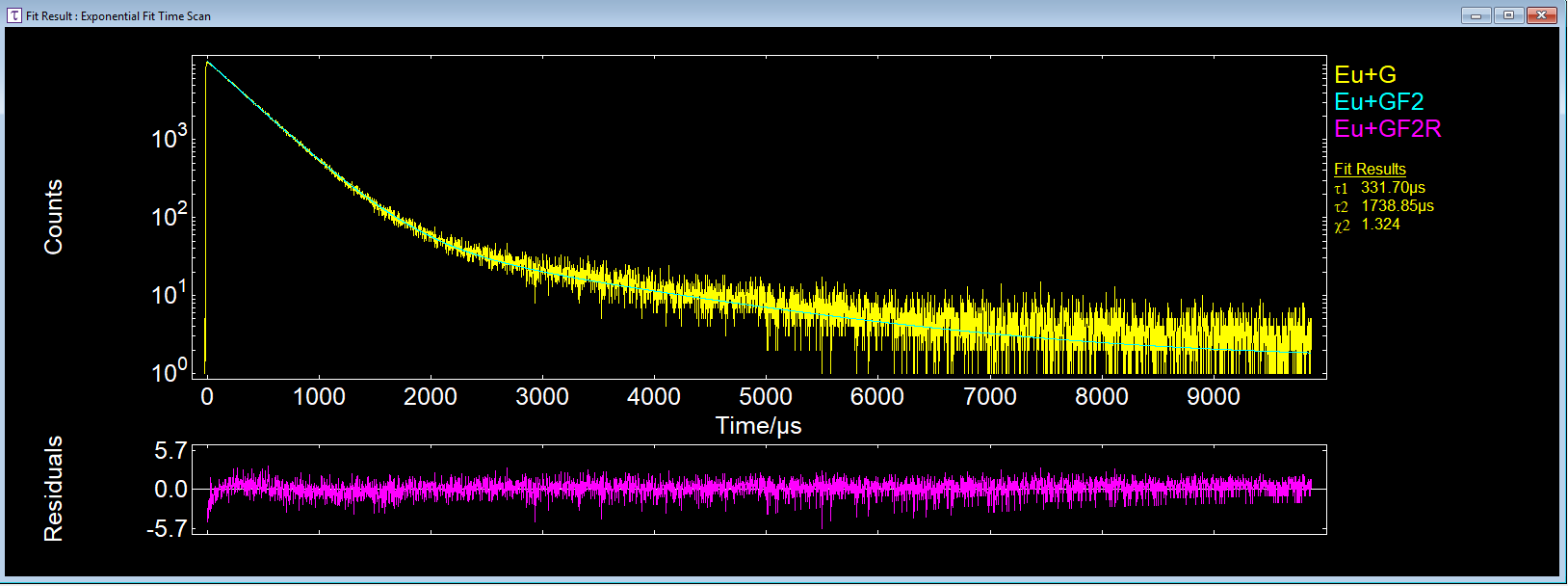
**

**Figure S30.** The fluorescence lifetime decay fitting curves of LP/Eu/DPA in aqueous solution (λ_ex_ = 254 nm, λ_em_ = 616 nm).

**
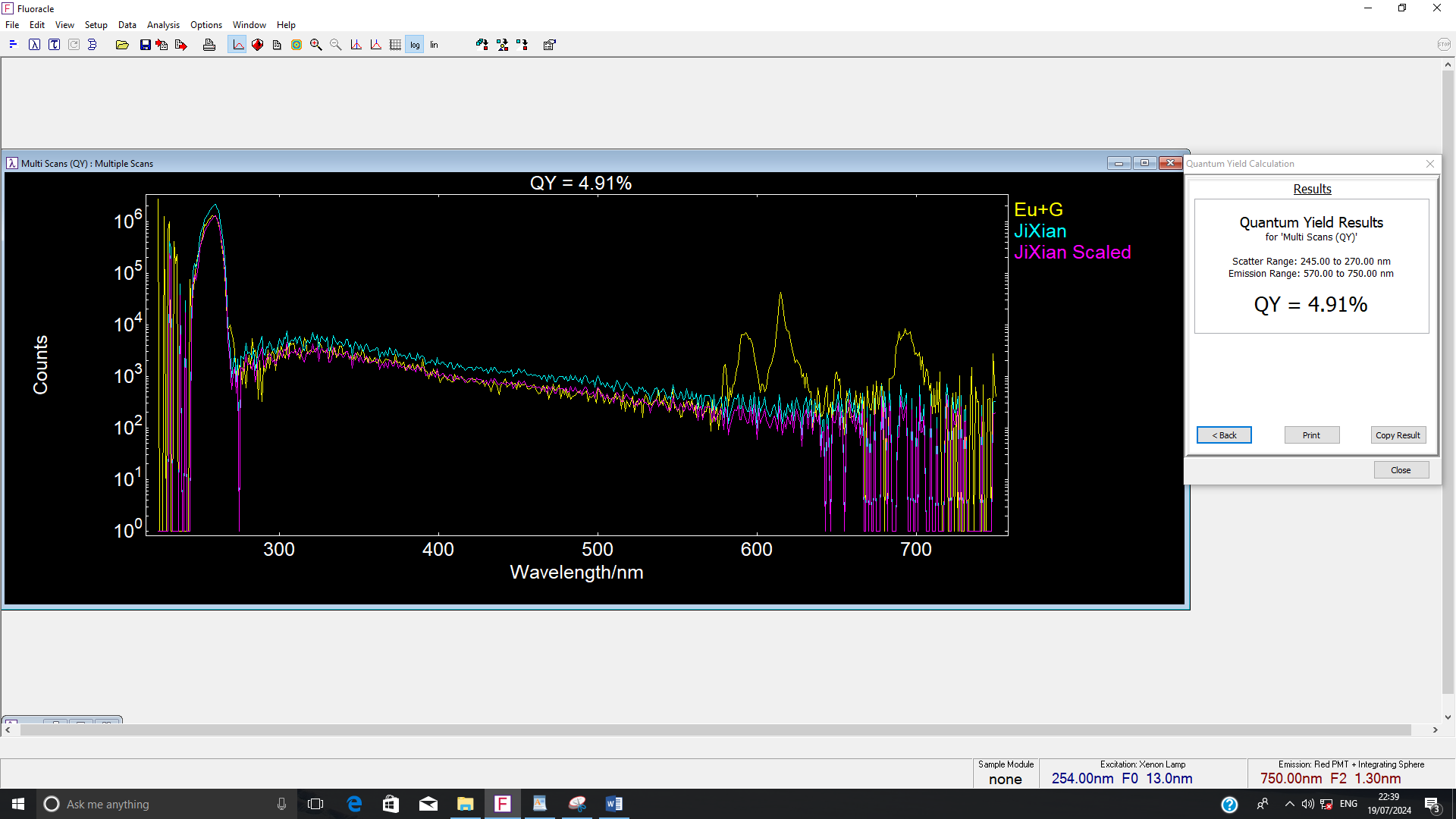
**

**Figure S31.** The fluorescence quantum yields of LP/Eu/DPA in aqueous solution (The value ranges from 570 nm to 750 nm, λ_ex_ = 300 nm).

**
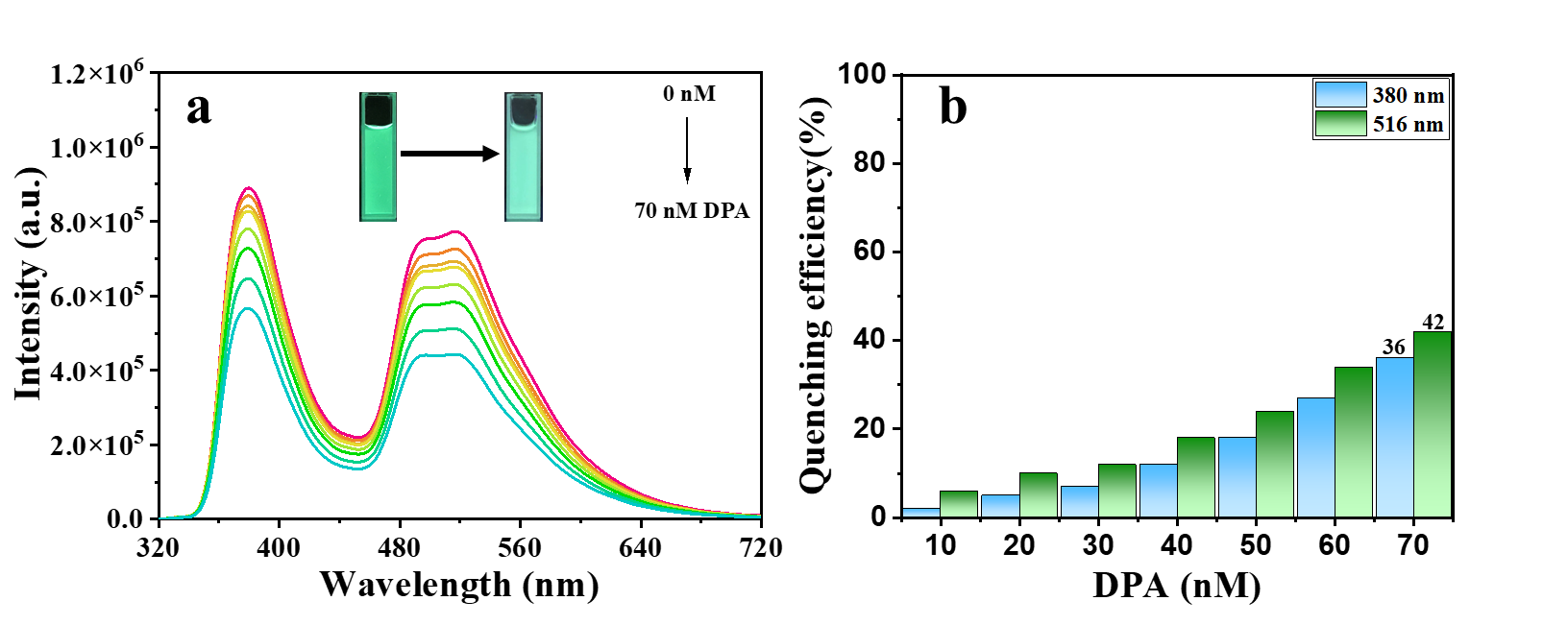
**

**Figure S32.** The prompt photoluminescence spectra of (a) DPA added into G aqueous solution. (b) The luminescent quenching efficiency for the emission of the G/LP with the DPA at 380 nm and 516 nm. ([G] = 0.01 mM, LP = 2 wt%, [DPA] = 70 nM).

**Figure S33.** The luminescent quenching efficiency for the emission of the G/LP/Eu with the DPA at 380 nm and 516 nm. ([G] = 0.01 mM, LP/Eu = 0.2 wt%, [DPA] = 60 nM).

**Figure S34.** (a) The energy gap between S_1_ and T_1_ in the presence of background charge with different quantity; (b) Schematic diagram of the change in the energy gap between S_1_ and T_1_ with the quantity of background charge; (c) Locations of the added background charge, electrostatic potential diagrams of the molecule and the charge distribution of key atoms in the molecule, under different quantity of background charges; (d) optimized geometries and relative Gibbs free energies of molecule G, under different quantity of background charges. The pink numbers are the quantities of the background negative charges.

**REFERENCES**

[1] (a) Y. Zhao, D. G. Truhlar, *Theor. Chem. Acc.* **2008**, *120*, 215-241; (b) P. C. Hariharan, J. A. Pople, *Theor. Chim. Acta.* **1973**, *28*, 213-222; (c) J. Tomasi, B. Mennucci, R. Cammi, *Chem. Rev.* **2005**, *105*, 2999; (d) S. Grimme, S. Ehrlich, L. Goerigk, *J. Comput. Chem.* **2011**, *32*, 1456-1465; (e) A. D. Laurent, D. Jacquemin, *Int. J. Quantum. Chem.* **2013**, *113*, 2019-2039; (f) M. J. Frisch, G. W. Trucks, H. B. Schlegel, G. E. Scuseria, M. A. Robb, J. R. Cheeseman, G. Scalmani, V. Barone, G. A. Petersson, H. Nakatsuji, X. Li, M. Caricato, A. V. Marenich, J. Bloino, B. G. Janesko, R. Gomperts, B. Mennucci, H. P. Hratchian, J. V. Ortiz, A. F. Izmaylov, J. L. Sonnenberg, D. Williams-Young, F. Ding, F. Lipparini, F. Egidi, J. Goings, B. Peng, A. Petrone, T. Henderson, D. Ranasinghe, V. G. Zakrzewski, J. Gao, N. Rega, G. Zheng, W. Liang, M. Hada, M. Ehara, K. Toyota, R. Fukuda, J. Hasegawa, M. Ishida, T. Nakajima, Y. Honda, O. Kitao, H. Nakai, T. Vreven, K. Throssell, J. A. Montgomery, J. E. Jr., Peralta, F. Ogliaro, M. J. Bearpark, J. J. Heyd, E. N. Brothers, K. N. Kudin, V. N. Staroverov, T. A. Keith, R. Kobayashi, J. Normand, K. Raghavachari, A. P. Rendell, J. C. Burant, S. S. Iyengar, J. Tomasi, M. Cossi, J. M. Millam, M. Klene, C. Adamo, R. Cammi, J. W. Ochterski, R. L. Martin, K. Morokuma, O. Farkas, J. B. Foresman, D. J. Fox, Gaussian 16, Revision C.02; Gaussian, Inc.: Wallingford, CT, 2019; (g) T. Lu, F. Chen, *J. Comput. Chem.* **2012**, *33*, 580-592; (h) T. Lu, *J. Chem. Phys.* **2024**, *161*, 082503.

The Cartesian Coordinates of the stationary points discussed in the text.

G(0)

Atom X Y Z

C 3.68392600 -0.05756600 0.09788000

C 2.93620300 -1.22913900 0.03600100

C 1.55846700 -1.14176000 -0.11738500

C 0.92689400 0.10542400 -0.19852200

C 1.70238900 1.26933300 -0.12901800

C 3.08184100 1.19377700 0.01417900

H 3.42274800 -2.19469700 0.11097000

H 0.97332800 -2.05556700 -0.14290000

H 1.23590100 2.24535300 -0.21712200

H 3.68242200 2.09497400 0.05548400

C -0.53780900 0.19314500 -0.35550500

C -1.25341200 -0.77747900 -1.07074700

C -1.26654300 1.25296100 0.20329000

C -2.62006900 -0.66905300 -1.20499900

H -0.75173200 -1.60844300 -1.55122000

C -2.63205600 1.31580000 0.03660900

H -0.78129700 2.02389900 0.78920000

N -3.28794800 0.36726000 -0.66104700

H -3.21427800 -1.38963500 -1.75360600

H -3.23747600 2.11233900 0.45195900

Br 5.56126600 -0.16823200 0.29958600

C -4.76489700 0.43116300 -0.77097500

C -5.42682700 -0.24549800 0.42339900

H -5.03692300 -0.05441300 -1.70988100

H -5.03616200 1.48623600 -0.84111800

C -6.94766700 -0.18686100 0.31011600

H -5.10871000 -1.29166900 0.48375800

H -5.10487500 0.25213800 1.34688400

H -7.26337900 0.86170900 0.19206400

H -7.26155700 -0.71924700 -0.59437000

N -7.54798500 -0.85720800 1.46349400

H -7.39552300 -0.28369700 2.29195700

H -8.55788900 -0.88702200 1.33935900

G(-0.25)

Atom X Y Z

C -3.67687200 0.10126100 0.06927600

C -2.91656400 1.20442200 -0.30532100

C -1.54051200 1.06276900 -0.43073600

C -0.92318700 -0.16757900 -0.17395900

C -1.71078300 -1.26078400 0.20724100

C -3.08893600 -1.13343100 0.32492900

H -3.39192900 2.16050200 -0.49114000

H -0.94517000 1.92924300 -0.70086100

H -1.25477500 -2.22953100 0.38541500

H -3.69939800 -1.98324500 0.60723600

C 0.54027300 -0.30839200 -0.30091600

C 1.26100600 0.41941000 -1.25832100

C 1.26267800 -1.17456900 0.53191600

C 2.62757100 0.27437400 -1.35136000

H 0.76371000 1.08382600 -1.95432100

C 2.62898400 -1.28343600 0.39638000

H 0.77255400 -1.75062100 1.30715000

N 3.29080300 -0.56749200 -0.53421300

H 3.22534600 0.81092700 -2.07801000

H 3.23042100 -1.93130900 1.02254500

Br -5.55206200 0.28435600 0.23572800

C 4.76933800 -0.64991500 -0.60660700

C 5.41127800 0.37022900 0.32637000

H 5.05058400 -0.47811700 -1.64731600

H 5.04724300 -1.67187100 -0.34241300

C 6.93383300 0.29232400 0.25961200

H 5.08888100 1.38099000 0.05442000

H 5.07592600 0.18163200 1.35394100

H 7.25499200 -0.73769500 0.48091400

H 7.26133300 0.51766900 -0.76114300

N 7.51142900 1.29549500 1.15420900

H 7.34022200 1.01215100 2.11799500

H 8.52379500 1.28893400 1.04795200

2.72039971 -3.96490368 -5.46125848 -0.25

G(-0.5)

Atom X Y Z

C -3.66115400 0.11476100 0.09379700

C -2.89724500 1.20952300 -0.29783400

C -1.52501900 1.05438400 -0.44767000

C -0.91519400 -0.18106400 -0.19768600

C -1.70622300 -1.26617300 0.19915200

C -3.08086500 -1.12536800 0.34072400

H -3.36674400 2.16972000 -0.47687000

H -0.92591900 1.91438800 -0.72990000

H -1.25575300 -2.23840800 0.37249800

H -3.69426100 -1.96853000 0.63625600

C 0.54597800 -0.33191800 -0.33909000

C 1.26178300 0.38051100 -1.31147200

C 1.27145900 -1.18817600 0.50124600

C 2.62902100 0.23888700 -1.40444700

H 0.76055200 1.03356400 -2.01547400

C 2.63783400 -1.29366800 0.36576400

H 0.78400200 -1.75471900 1.28507800

N 3.29649600 -0.58549500 -0.57320100

H 3.22422100 0.76821600 -2.13844300

H 3.24266400 -1.92943700 1.00112900

Br -5.53037500 0.31850600 0.29850200

C 4.77689600 -0.64806300 -0.62791200

C 5.38715300 0.37346700 0.32539400

H 5.06994300 -0.46257700 -1.66295200

H 5.06544000 -1.66845800 -0.36881600

C 6.91167300 0.31436800 0.29942300

H 5.05964300 1.38197600 0.05136900

H 5.02836800 0.17555000 1.34334900

H 7.23955900 -0.71354200 0.52055900

H 7.26488500 0.55345300 -0.70950300

N 7.45066100 1.31632700 1.21942000

H 7.25692700 1.02101100 2.17534300

H 8.46557300 1.32498200 1.14122400

2.72039971 -3.96490368 -5.46125848 -0.5

G(-0.75)

Atom X Y Z

C -3.63643300 0.13606800 0.12695600

C -2.86537500 1.21980700 -0.28073800

C -1.49941000 1.04418900 -0.46276700

C -0.90310400 -0.20070200 -0.22774900

C -1.70097800 -1.27496100 0.18412000

C -3.06988900 -1.11375700 0.35676300

H -3.32428900 2.18759100 -0.44627400

H -0.89305700 1.89549100 -0.75612100

H -1.26049500 -2.25379100 0.34650300

H -3.68919500 -1.94827800 0.66443000

C 0.55435900 -0.36774400 -0.38887200

C 1.26446500 0.32459900 -1.37957700

C 1.28259600 -1.21371900 0.45940000

C 2.63250800 0.18629400 -1.47258500

H 0.75877300 0.96462900 -2.09236100

C 2.64903900 -1.31540800 0.32427800

H 0.79777400 -1.76882000 1.25312800

N 3.30457800 -0.61573500 -0.62349500

H 3.22519800 0.70667800 -2.21507700

H 3.25755600 -1.93653400 0.97053800

Br -5.49655500 0.36910700 0.37869400

C 4.78693400 -0.64982800 -0.65341800

C 5.35169600 0.37937300 0.32094300

H 5.09529800 -0.45154700 -1.68165900

H 5.09132700 -1.66585700 -0.39473300

C 6.87684200 0.34879000 0.35261200

H 5.01597500 1.38267200 0.03804700

H 4.96141300 0.17361000 1.32585200

H 7.21589200 -0.67380500 0.58121400

H 7.26494600 0.60118300 -0.64003600

N 7.35894800 1.35535900 1.29931900

H 7.13394500 1.04975000 2.24513900

H 8.37574200 1.38525900 1.26143000

2.72039971 -3.96490368 -5.46125848 -0.75

G(-1.0)

Atom X Y Z

C -3.58202800 0.17995500 0.18833800

C -2.80048200 1.23771400 -0.26491000

C -1.44991900 1.02090900 -0.50941100

C -0.87937200 -0.23865900 -0.28930700

C -1.68807800 -1.28731900 0.16499000

C -3.04243000 -1.08537500 0.39814100

H -3.23826900 2.21774000 -0.41529400

H -0.83248600 1.85183100 -0.83653700

H -1.26735200 -2.27660600 0.31667600

H -3.67103800 -1.89949700 0.73979900

C 0.56994100 -0.43847700 -0.48563800

C 1.27088400 0.21038200 -1.51148700

C 1.30238600 -1.26141900 0.38148100

C 2.64079500 0.07917000 -1.60124000

H 0.75837100 0.82217000 -2.24415900

C 2.66919600 -1.35538000 0.25009800

H 0.82166900 -1.79178100 1.19445200

N 3.32041400 -0.67424500 -0.71445100

H 3.22973600 0.57866800 -2.36112600

H 3.28314000 -1.94581500 0.91966400

Br -5.41958300 0.46932900 0.53387600

C 4.80367200 -0.64844500 -0.69420200

C 5.27680800 0.38374900 0.32722700

H 5.14044100 -0.41264400 -1.70552900

H 5.14285200 -1.65607200 -0.44515500

C 6.79754800 0.42897700 0.44208100

H 4.90368800 1.37557700 0.05061500

H 4.84925400 0.13640900 1.30753900

H 7.17803300 -0.58336600 0.64989000

H 7.22624600 0.74353800 -0.51531500

N 7.17066200 1.41788000 1.45517300

H 6.91288600 1.05899100 2.37361400

H 8.18471300 1.50461200 1.47574200

2.72039971 -3.96490368 -5.46125848 -1.0
